# Long noncoding RNA SNHG12 induces proliferation, migration, epithelial–mesenchymal transition, and stemness of esophageal squamous cell carcinoma cells via post‐transcriptional regulation of BMI1 and CTNNB1

**DOI:** 10.1002/1878-0261.12683

**Published:** 2020-06-18

**Authors:** Duoguang Wu, Xiaotian He, Wenjian Wang, Xueting Hu, Kefeng Wang, Minghui Wang

**Affiliations:** ^1^ Guangdong Provincial Key Laboratory of Malignant Tumor Epigenetics and Gene Regulation Department of Thoracic Surgery Sun Yat‐sen Memorial Hospital Sun Yat‐sen University Guangzhou China

**Keywords:** BMI1, CTNNB1, esophageal squamous cell carcinoma, SNHG12

## Abstract

Esophageal squamous cell carcinoma (ESCC) is one of the most common malignant tumors around the world. Numerous studies have revealed the function of long noncoding RNAs (lncRNAs) in cancers, including ESCC. In this study, lncRNA small nucleolar RNA host gene 12 (SNHG12), mainly distributed in ESCC cell cytoplasm, was overexpressed in ESCC specimens and CD133^+^ cells. In CD133^‐^ ESCC cells, SNHG12 overexpression promoted cell proliferation, migration, epithelial–mesenchymal transition (EMT), and stemness and SNHG12 silencing led to opposite results. Furthermore, SNHG12 sequestered miR‐6835‐3p and induced the proto‐oncogene, polycomb ring finger (BMI1). SNHG12 also enhanced the stability of CTNNB1, the mRNA encoding β‐catenin, via recruiting insulin‐like growth factor 2 mRNA‐binding protein 2 (IGF2BP2) in ESCC. Rescue assays indicated that CTNNB1 and BMI1 were targets for SNHG12 to regulate ESCC cell proliferation, migration, EMT, and stemness. Furthermore, SOX4 (sex‐determining region Y‐box 4) bound with the SNHG12 promoter to transcriptionally activate SNHG12 in ESCC. Finally, *in vivo* data showed SNHG12 knockdown retarded tumorigenesis and metastasis in ESCC. In summary, SNHG12 induces proliferation, migration, EMT, and stemness of ESCC cells via post‐transcriptional regulation of BMI1 and CTNNB1, indicating that targeting SNHG12 might be a novel target for ESCC treatment.

## Graphical abstract

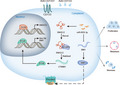

**Abbreviations**
AS: antisense
EMT: epithelial–mesenchymal transition
ESCA: esophageal carcinoma
ESCC: esophageal squamous cell carcinoma
RBP: RNA‐binding protein
RIP: RNA immunoprecipitation
RISC: RNA‐induced silencing complex
SNHG12: small nucleolar RNA host gene 12
SOX4: sex‐determining region Y‐box 4
TCGA: The Cancer Genome Atlas

## 1 Introduction

Esophageal squamous cell carcinoma (ESCC), one of the most common and major malignant tumors worldwide, is the main histological type of esophageal cancer, and its mortality rate is unexpectedly high (Jemal *et al.*, 2011). Clinically, prognosis in ESCC is poor with 5‐year survival less than 10% despite the great advancement in operative treatments. Besides, the median survival of ESCC patients in terminal stage is shorter than 1 year (Lv *et al.*, 2013; Rustgi and El‐Serag, 2014). Owing to the lack of specific symptoms in early cancer stage, most ESCC patients are usually diagnosed at the later stage with abundant regional invasion and local lymph node metastasis (Li *et al.*, 2016; Miyazaki *et al.*, 2003). Therefore, discovery of fresh and accurate early diagnostic molecular markers and better understanding of the important molecular mechanisms of ESCC are essential for improving the prognosis and survival of patients with ESCC.

Long noncoding RNAs (lncRNAs) belong to a group of evolutionarily conserved RNA molecules with a length of more than 200 nucleotides, which lack protein‐coding ability (Ma *et al.*, 2013). Numerous evidences indicate that lncRNAs play various functional roles in multiple kinds of biological processes, including cell growth, invasion, migration, and tumorigenesis (Gibb *et al.*, 2011; Huarte and Rinn, 2010). Additionally, aberrant expression of lncRNAs has been associated with the occurrence and progression of multiple tumors, including ESCC (Hao *et al.*, 2015; Li *et al.*, 2013). The oncogenic role of lncRNA small nucleolar RNA host gene 12 (SNHG12) has been verified in recent researches. For example, lncRNA SNHG12 contributes to the development of cervical cancer by regulating miR‐125b/STAT3 axis (Jin *et al.*, 2019). LncRNA SNHG12 is related to the poor prognosis of prostate cancer patients and facilitates tumorigenesis through sponging miR‐133b (Cheng *et al.*, 2019). LncRNA SNHG12 motivates cell proliferation and invasion in colorectal cancer via serving as the sponge of miR‐16 (Liu *et al.*, 2019). Nevertheless, the function of SNHG12 in ESCC is poorly comprehended.

The Wnt/β‐catenin pathway is implicated in many vital cellular functions such as maintaining cancer stem cells and facilitating epithelial‐to‐mesenchymal transition (EMT) (Krishnamurthy and Kurzrock, 2018; Le *et al.*, 2019). For instance, lncRNA SNHG16 promotes bladder cancer progression through modulating miR‐98/STAT3/Wnt/β‐catenin pathway axis (Feng *et al.*, 2018). LncRNA ZEB2‐AS1 stimulates the initiation of gastric cancer by activating the Wnt/β‐catenin pathway (Wang *et al.*, 2019a). Knockdown of lncRNA SNHG5 represses the development of glioma through Wnt/CTNNB1 signaling pathway (Hu *et al.*, 2019). Moreover, the interaction between SNHG12 and Wnt pathway has been researched in papillary thyroid carcinoma and prostate cancer (Ding *et al.*, 2018; Song *et al.*, 2019). Hence, it is worthy to explore the effects of SNHG12 on Wnt pathway downstream target in ESCC.

In this study, we planned to research the effects of SNHG12 in ESCC cell proliferation, migration, EMT, and stemness via post‐transcription regulation mechanism.

## 2 Materials and methods

### 2.1 Tissue collection

The primary ESCC specimens (*n* = 70) and the paired adjacent normal specimens (*n* = 70) were resected from 70 patients who were diagnosed with ESCC referring to histopathologic evaluation and received surgery at Sun Yat‐sen Memorial Hospital. Experiment protocols received approval from the Ethics Committee of Sun Yat‐sen Memorial Hospital, and the signed informed consents were collected from all ESCC patients. No participants underwent any other treatment before surgery. The study methodologies conformed to the standards set by the Declaration of Helsinki.

### 2.2 Cell culture and treatment

Human ESCC cell lines including EC9706, EC109, KYSE410, KYSE150, and KYSE450 were all available from the ATCC (Manassas, VA, USA) for cell culture with 5% CO_2_ at 37 °C. RPMI‐1640 medium (Gibco, Carlsbad, CA, USA) was acquired commercially, with 1% Pen/Strep mixture and 10% FBS as supplements. Medium was changed every 3 days. After cells had reached about 80% confluence at the 3rd passage, CD133^+^ cancer cells were obtained by treating with MACS CD133 kit (Miltenyi Biotec, Teterow, Germany). ESCC cells without treatment were termed CD133^‐^ cancer cells as control. About 2 mg·mL^−1^ of actinomycin D was procured from Sigma‐Aldrich (St. Louis, MO, USA) to treat cells.

### 2.3 Total RNA extraction and quantitative real‐time polymerase chain reaction

Total RNA extraction was performed in cells using TRIzol method (Invitrogen, Carlsbad, CA, USA). The synthesis of cDNA template was achieved based on instruction of PrimeScript RT Reagent Kit (Takara, Shiga, Japan). After that, the SYBR^®^ Premix Ex Taq™ II (Takara) was used to prepare the PCR system. Using 2^−ΔΔCt^ method, data normalization was performed and relative to U6 or GAPDH.

### 2.4 Subcellular fractionation

Using PARIS™ Kit (Ambion, Austin, TX, USA), the nucleus or cytoplasm of CD133^‐^EC109/KYSE410 and CD133^+^EC109/KYSE410 cell samples was severally separated as per instruction. The isolated SNHG12 was assayed by quantitative real‐time polymerase chain reaction (qRT‐PCR), with GAPDH and U6 as indictors.

### 2.5 Fluorescence in situ hybridization

The specific fluorescence in situ hybridization (FISH) probe of SNHG12 was produced by RiboBio (Guangzhou, China) and employed according to user manual. After staining nuclei with Hoechst solution, stained cell samples were visualized with Olympus fluorescence microscope (Tokyo, Japan).

### 2.6 Plasmid transfection

The specific shRNAs and control shRNAs were available from GenePharma (Shanghai, China) to silence SNHG12, BMI1, IGF2BP2, CTNNB1, SOX2, and SOX4 in processed cells using Lipofectamine 2000 (Invitrogen). Besides, the pcDNA3.1/SNHG12, pcDNA3.1/BMI1, pcDNA3.1/IGF2BP2, pcDNA3.1/IGF2BP3, pcDNA3.1/SOX2, pcDNA3.1/SOX4, and their relative control pcDNA3.1 vectors were all procured from GeneChem (Shanghai, China). The miR‐6835‐3p mimics and NC mimics were also from GenePharma. After 48 h of transfection, cells were reaped.

### 2.7 Colony formation

Clonogenic cells from various groups were plated at 500 cells per well into 6‐well plates for the 14‐day incubation. Thereafter, cells were fixed with 4% paraformaldehyde and stained with 0.1% crystal violet for counting.

### 2.8 EdU assay

Processed cells of EC109 and KYSE410 were seeded in 96‐well plates for EdU assay by using the EdU detection kit from RiboBio. Cells were visualized under fluorescent microscope after adding Hoechst 33342 solution.

### 2.9 Transwell migration and invasion assay

1 × 10^5^ cells from each group were placed into the upper chamber inserted in Transwell apparatus (Corning Incorporated, Corning, NY, USA), precoated with (for invasion) or without (for migration) Matrigel (BD Biosciences). Lower chamber was supplemented with complete medium. Migrating cells to the bottom were stained after 24 h in 0.5% crystal violet for observation with microscope.

### 2.10 Western blot

Total protein samples were prepared using RIPA lysis buffer on ice, and then, 50 μg of protein was separated on 12% SDS/PAGE, shifted to PVDF membranes. After culture with 5% skimmed milk, membranes were probed all night with the diluted primary antibody against internal control GAPDH or vimentin, N‐cadherin, E‐cadherin, SOX2, SOX4, OCT4, Nanog, C‐myc, MMP7, and β‐catenin. The HRP‐labeled secondary antibody was used after washing in TBST. All antibodies were available from Abcam (Cambridge, MA, USA). Proteins were finally subjected to ECL Prime Western Blotting Detection Reagent (GE Healthcare, Chicago, IL, USA).

### 2.11 Sphere formation assay

Cells at 10 cells/well were placed to the 96‐well ultralow attachment plates (Corning Inc.) adding sphere medium for 7‐day incubation. Sphere cells with diameter> 50 mm were counted. Sphere formation efficiency equaled to sphere number/seeded cell number × 100%, and the control group was set as 1.

### 2.12 Flow cytometry analysis

To sort CD133 + ESCC cells, phosphate‐buffered saline (PBS; Sigma‐Aldrich) was applied to wash cells and then bovine serum albumin (BSA; Sigma‐Aldrich) was used to fix. Thereafter, cells were stained with FITC mouse anti‐CD133 (Abcam) and sorted by the FACSAria II instrument (BD Biosciences, Franklin Lakes, NJ, USA).

### 2.13 RNA immunoprecipitation assay

RNA immunoprecipitation (RIP) assay was implemented as per the instruction of Magna RIP™ RNA‐Binding Protein Immunoprecipitation Kit (Millipore, Bedford, MA, USA). Magnetic beads were bound to human Ago2 antibody or IGF2BP2 antibody at room temperature for 1 h. Normal mouse IgG antibody was taken as control. Following immunoprecipitation, RNA enrichment was assayed by qRT‐PCR.

### 2.14 Pull‐down assay

For RNA pull‐down, protein extracts from different cell samples were mixed with bead‐bound biotinylated SNHG12 probes or control probes. For DNA pull‐down, DNA pull‐down test kit (Gzscbio, Guangzhou, China) was employed as per instruction. SNHG12 promoter was amplified by PCR, biotinylated, and bound with beads. The nonbiotinylated promoter was taken as control probe. Both pull‐downs were analyzed for detecting RNA enrichment.

### 2.15 Luciferase reporter assay

SNHG12 or BMI1 fragments covering miR‐6835‐3p wild‐type and mutant binding sequences were used to construct reporter vectors with luciferase reporter pmirGLO (Promega, Madison, WI, USA). The acquired SNHG12‐WT/Mut and BMI1‐WT/Mut were cotransfected with indicated transfection plasmids in HEK 293T cells. Besides, the wild‐type or mutant SOX4 binding sites to SNHG12 promoter were cloned to pGL3 vector (Promega) and then transfected into CD133^+^EC109 or CD133^‐^EC109 cells. All of the luciferase activities were monitored using Luciferase Reporter Assay System (Promega).

### 2.16 Chromatin immunoprecipitation assay

After cross‐linking protein and DNA, samples were randomly fragmented to 200‐1000 bp by ultrasonic for immunoprecipitation with target protein‐specific antibody to SOX4. Anti‐IgG antibody served as control. The binding of SOX4 to SNHG12 was assessed by qRT‐PCR.

### 2.17 Animal assays

The BALB/c nude mice (male, 4–5 weeks old) were provided by the Shanghai Experimental Animal Center of the Chinese Academy of Sciences. All mice were kept with sterilized water and food. CD133^+^ EC109 cells (3 × 10^6^) transfected with sh‐NC or sh‐SNHG13#1 were injected into each mouse subcutaneously. The tumor volume (length × width^2^×0.5.) of mice was examined every 4 days. Mice were sacrificed through cervical dislocation after 28 days, and weight of tumors was evaluated.

To establish metastasis model, the CD133^+^ EC109 cells transfected with sh‐NC or sh‐SNHG13#1 were injected from the tail vein of mice. After killing the mice, lungs were removed and subjected to paraffin embedding. The consecutive sections (4 μm) underwent hematoxylin and eosin (HE) staining. The metastatic nodules were counted. All experiments were performed referring to the Guide for the Care and Use of Laboratory Animals of the National Institutes of Health. Protocols received the approval of the committee of animal experimentation of Sun Yat‐sen Memorial Hospital.

### 2.18 Immunohistochemistry

Mouse tissues were paraffin‐embedded, and level of Ki‐67 and PCNA was examined by Immunohistochemistry (IHC). Each section was treated with 3% H_2_O_2_ as well as 5% BSA and was incubated with the primary antibodies against Ki‐67 and PCNA (Abcam) for 12 h at 4 °C, followed by incubation with the HRP‐conjugated secondary antibody for 1 h under 37 °C. After washing the sections, sections were counterstained using hematoxylin and observed under the microscope (Olympus, Japan).

### 2.19 Statistical analyses

Results were all exhibited as the mean ± SD of independent bio‐triplicates, with data analysis using prism 5.0 software (GraphPad Software, Inc., La Jolla, CA, USA). The *P*‐value was calculated by Student’s *t*‐test for two groups or one‐way ANOVA for multiple groups, with values below 0.05 indicating statistical significance.

## 3 Results

### 3.1 SNHG12 expression is upregulated in ESCC tissues and cells

**Fig. 1:**
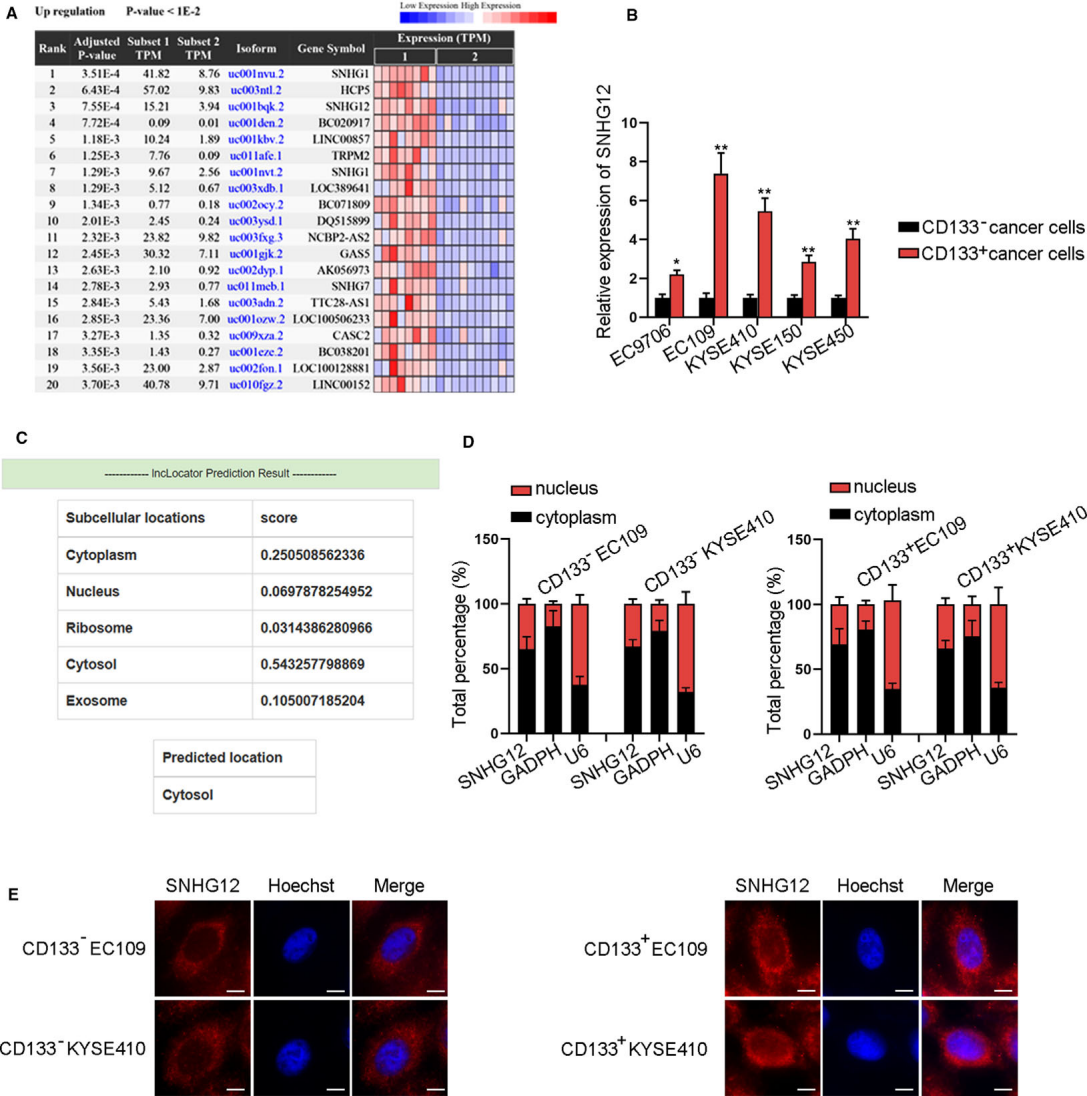
SNHG12 expression is upregulated in ESCC tissues and cells. (A) The upregulated lncRNAs in ESCA (esophageal carcinoma) tissues were obtained by Cancer RNA‐Seq Nexus analysis. (B) qRT‐PCR of SNHG12 level in CD133^+^ cancer cells and CD133^‐^ cancer cells (*n* = 5; Student’s *t*‐test). (C) lncLocator predicted that SNHG12 expression was mainly distributed in cytoplasm. (D,E) Subcellular fraction (*n* = 5; Student’s *t*‐test) and FISH (bar value = 20 μm) assays measured the distribution of SNHG12 in ESCC cells. Results were all exhibited as the mean ± standard deviation (SD) and taken from more than three independent experiments. *P < 0.05, **P < 0.01

First, to confirm the regulatory pattern of SNHG12 in ESCC, we found that SNHG1, SNHG7, and SNHG12 were three upregulated lncRNAs in ESCC tissues through Cancer RNA‐Seq Nexus analysis (Fig. 1A). The carcinogenesis function of SNHG family in cancers has been widely reported (Cai *et al.*, 2017; Dong *et al.*, 2018), and the participation of SNHG1 and SNHG7 in ESCC has been researched (Xu *et al.*, 2018a; Zhang *et al.*, 2017), so we focused on SNHG12 in our research. Then, qRT‐PCR data depicted that SNHG12 was higher in ESCC tissues than paired para‐tumor specimens (Fig. S1A). According to Kaplan–Meier analysis, high SNHG12 level was linked to unsatisfactory survival in ESCC patients (Fig. S1B), indicating that SNHG12 was a prognostic marker in ESCC. Through analyzing the pathological features in ESCC specimens, we showed that SNHG12 expression was related to tumor size (*P* = 0.001), TNM stage (*P* = 0.016), and lymph node metastasis (*P *= 0.003) in ESCC patients (Table 1), indicating that SNHG12 potentially participated in tumorigenesis and metastasis in ESCC.

**Table 1:** Correlation between SNHG12 Expression and Clinical Features of ESCC (n = 70).

Variable	SNHG12		*P*‐value
	Low	High	
Age			
<60	19	17	0.811
≥60	16	18	
Gender			
Male	12	9	0.602
Female	23	26	
Smoking status			
No	11	9	0.791
Yes	24	26	
Tumor size			
<3cm	24	8	0.001**
≥3cm	11	27	
TNM			
I–II	22	11	0.016**
III–IV	13	24	
Lymph node metastasis			
No	23	10	0.003**
Yes	12	25	
Differentiation			
Moderate and high	14	12	0.804
Low	21	23	

Low/high expression groups were divided by the sample median. Pearson’s chi‐square test. *P < 0.05, **P < 0.01 were considered to be statistically significant.

To explore the relation between SNHG12 and stem cell‐like properties in ESCC cells, we sorted CD133^+^ cells from 5 ESCC cell lines using flow cytometry (Fig. S2A). Then, we enriched the stem cell‐like ESCC cells through sphere formation assay. Flow cytometry confirmed that CD133 + cell ratio in ESCC cell‐derived spheres was higher than parental ESCC cells (Fig. S2B). The levels of stem‐like cell‐specific markers (SOX2, SOX4, OCT4, and Nanog) were higher in tumorspheres than in parental ESCC cells (Fig. S2C). Therefore, CD133+ cells sorted from the tumor spheres were used in subsequent assays, and the parental ESCC cells, named as CD133‐ cells, were taken as control cells. According to qRT‐PCR data, SNHG12 expression was obviously upregulated in CD133^+^ ESCC cells compared with the CD133^‐^ cancer cell group. CD133^+^EC109 and CD133^+^KYSE410 cells expressed the highest SNHG12 expression (Fig. 1B). Next, bioinformatics tool lncLocator predicted that SNHG12 expression is mainly distributed in cytoplasm (Fig. 1C), and this was verified by subcellular fraction and FISH assays (Fig. 1D,E). Additionally, we could see that the FISH intensity of SNHG12 was stronger in CD133^+^ ESCC cells than in CD133^‐^ ESCC cells (Fig. 1E). Totally, SNHG12 was upregulated in ESCC tissues and cells, and mainly distributed in cytoplasm.

### 3.2 SNHG12 upregulation promotes cell proliferation, migration, and EMT as well as cell stemness in ESCC

**Fig. 2:**
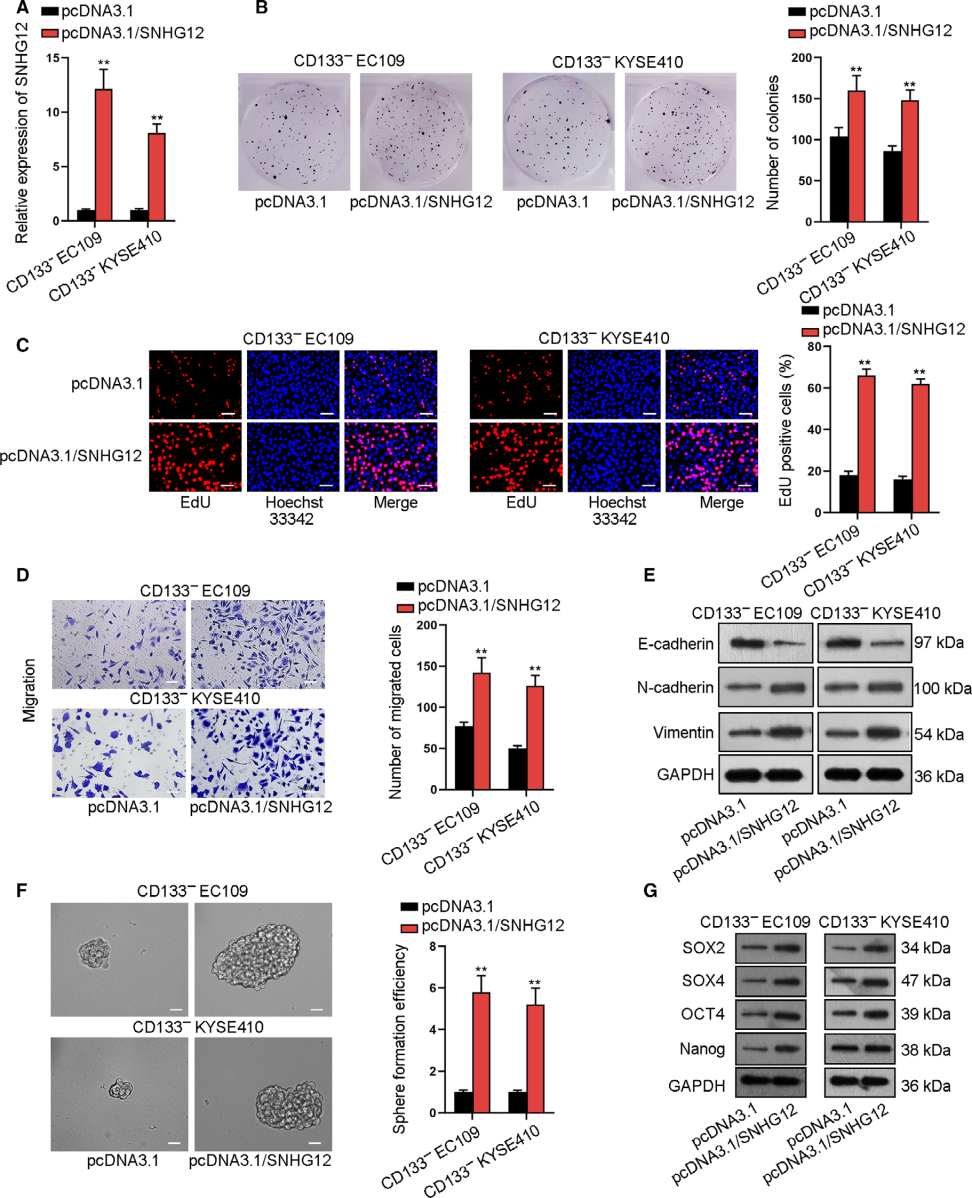
SNHG12 upregulation promotes cell proliferation, migration, and EMT as well as cell stemness in ESCC. (A) qRT‐PCR confirmed SNHG12 overexpression by pcDNA3.1/SNHG12 (*n* = 5; Student’s *t*‐test). (B‐C) Colony formation and EdU (bar value = 100 μm) assays detected ESCC cell proliferation when cells were transfected with pcDNA3.1/SNHG13 (*n* = 5; Student’s *t*‐test). (D) Transwell assay measured ESCC cell migration ability when overexpressing SNHG12 (bar value = 100 μm; *n* = 5; Student’s *t*‐test). (E) Western blot assay tested the expression of EMT‐related proteins (E‐cadherin, N‐cadherin, and vimentin; *n* = 5). (F) Sphere formation assay detected the ESCC cell sphere formation efficiency (bar value = 100 μm; *n* = 5; Student’s *t*‐test). (G) Western blot assay measured the protein expression of stem cell markers (SOX2, SOX4, OCT4, and Nanog; *n* = 5). Results were all exhibited as the mean ± standard deviation (SD) and taken from more than three independent experiments. **P < 0.01

To comprehend the impact of SNHG12 in ESCC, we confirmed a satisfactory overexpression efficiency of SNHG12 in CD133^‐^EC109 and CD133^‐^KYSE410 cells (Fig. 2A). Subsequently, several gain‐of‐function assays were employed. SNHG12 upregulation increased colonies and EdU‐stained ESCC cells, indicating that SNHG12 facilitated proliferation of CS133^‐^ ESCC cells (Fig. 2B,C). Transwell assays explained that cell migration and invasion were expedited by SNHG12 overexpression (Fig. 2D and S3A). The EMT (epithelial**–**mesenchymal transition) markers were also tested. SNHG12 upregulation increased N‐cadherin and vimentin levels, but lessened E‐cadherin level (Fig. 2E). Additionally, sphere formation assay illustrated that upregulating SNHG12 strengthened the sphere formation efficiency of ESCC cells (Fig. 2F). Flow cytometry showed the increase of CD133^+^ cell ratio in ESCC cells under SNHG12 overexpression (Fig. S3B). Western blot assay also tested the protein expression of stem cell markers (SOX2, SOX4, OCT4, and Nanog) was increased when overexpressing SNHG12 (Figs 2G and S3C). In the whole, SNHG12 upregulation promoted cell proliferation, migration, and EMT as well as cell stemness in ESCC.

### 3.3 SNHG12 downregulation suppresses cell proliferation, migration, and EMT as well as cell stemness in ESCC

**Fig. 3:**
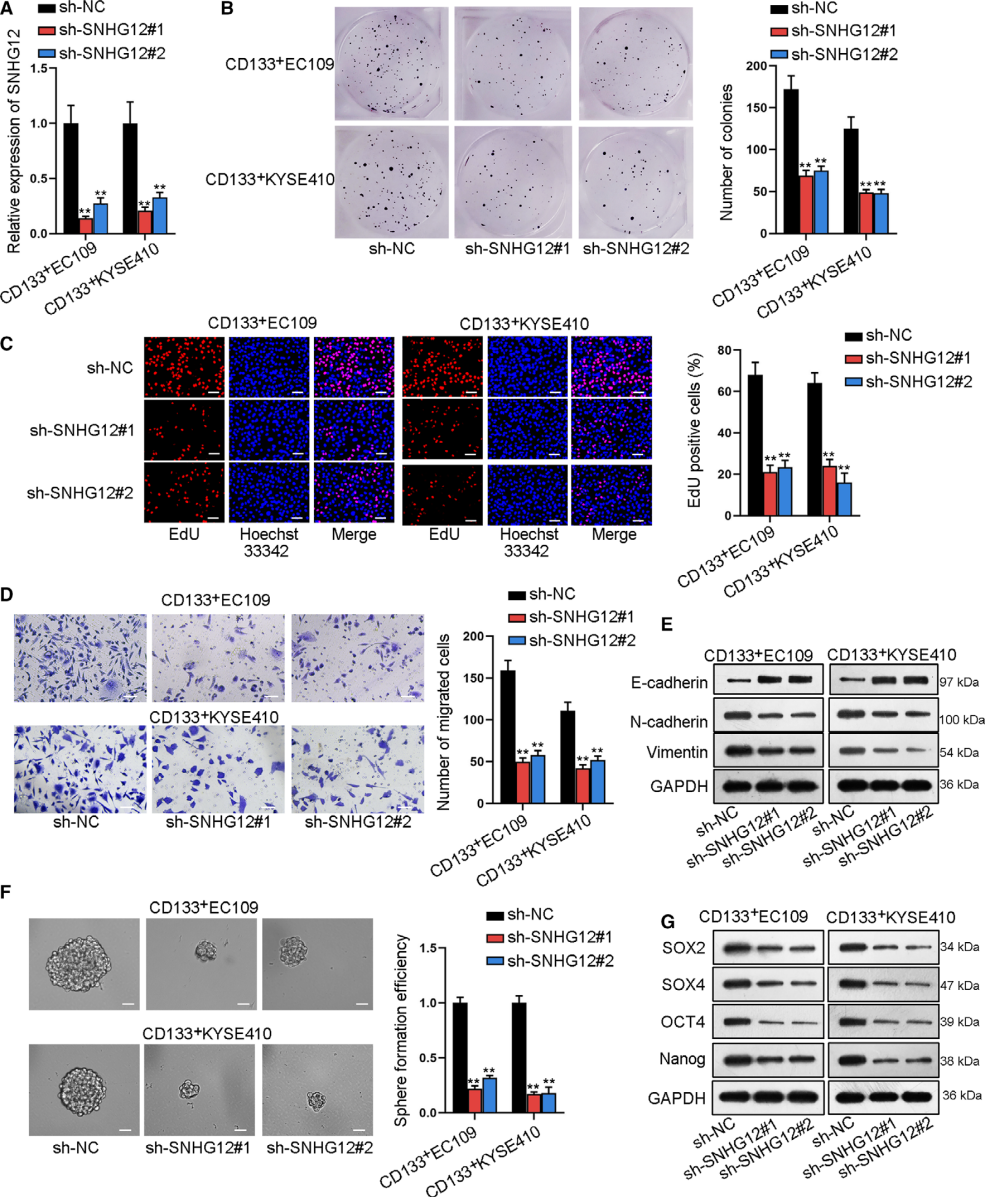
SNHG12 downregulation suppresses cell proliferation, migration, and EMT and strengthens cell stemness in ESCC. (A) qRT‐PCR validated SNHG12 knockdown by sh‐SNHG12#1/2 (*n* = 5; one‐way ANOVA). (B‐C) Colony formation and EdU (bar value = 100 μm) assays detected ESCC cell proliferation under SNHG12 knockdown (*n* = 5; one‐way ANOVA). (D) Transwell assay measured ESCC cell migration ability when inhibiting SNHG12 (bar value = 100 μm; *n* = 5; one‐way ANOVA). (E) Western blot assay tested the expression of EMT‐related proteins (E‐cadherin, N‐cadherin, and vimentin; *n* = 5). (F) Sphere formation assay detected the ESCC cell sphere formation efficiency (bar value = 100 μm; *n* = 5; one‐way ANOVA). (G) Western blot assay measured the protein expression of stem cell markers (SOX2, SOX4, OCT4, and Nanog; *n* = 5). Results were all exhibited as the mean ± standard deviation (SD) and taken from more than three independent experiments. **P < 0.01

Furthermore, loss‐of‐function assays were carried out in CD133^+^EC109 and CD133^+^KYSE410 cells. First, a pleasing knockdown efficiency of SNHG12 was obtained (Fig. 3A). Colony formation and EdU experiments depicted that SNHG12 downregulation suppressed cell proliferation (Fig. 3B,C). Transwell and western blot demonstrated that cell migration, invasion, and EMT process were hindered by silencing SNHG12 (Fig. 3D,E and S4A). Moreover, sphere formation efficiency declined and levels of stem cell markers (SOX2, SOX4, OCT4, and Nanog) were reduced in sh‐SNHG12#1/#2‐transfected ESCC cells (Fig. 3F,G). CD133^+^ ratio in ESCC cells decreased under SNHG12 knockdown (Fig. S4B). In a summary, SNHG12 downregulation suppressed cell proliferation and migration, retarded EMT, and weakened cell stemness in ESCC cells.

### 3.4 SNHG12 upregulates BMI1 expression via sequestering miR‐6835‐3p in ESCC

**Fig. 4:**
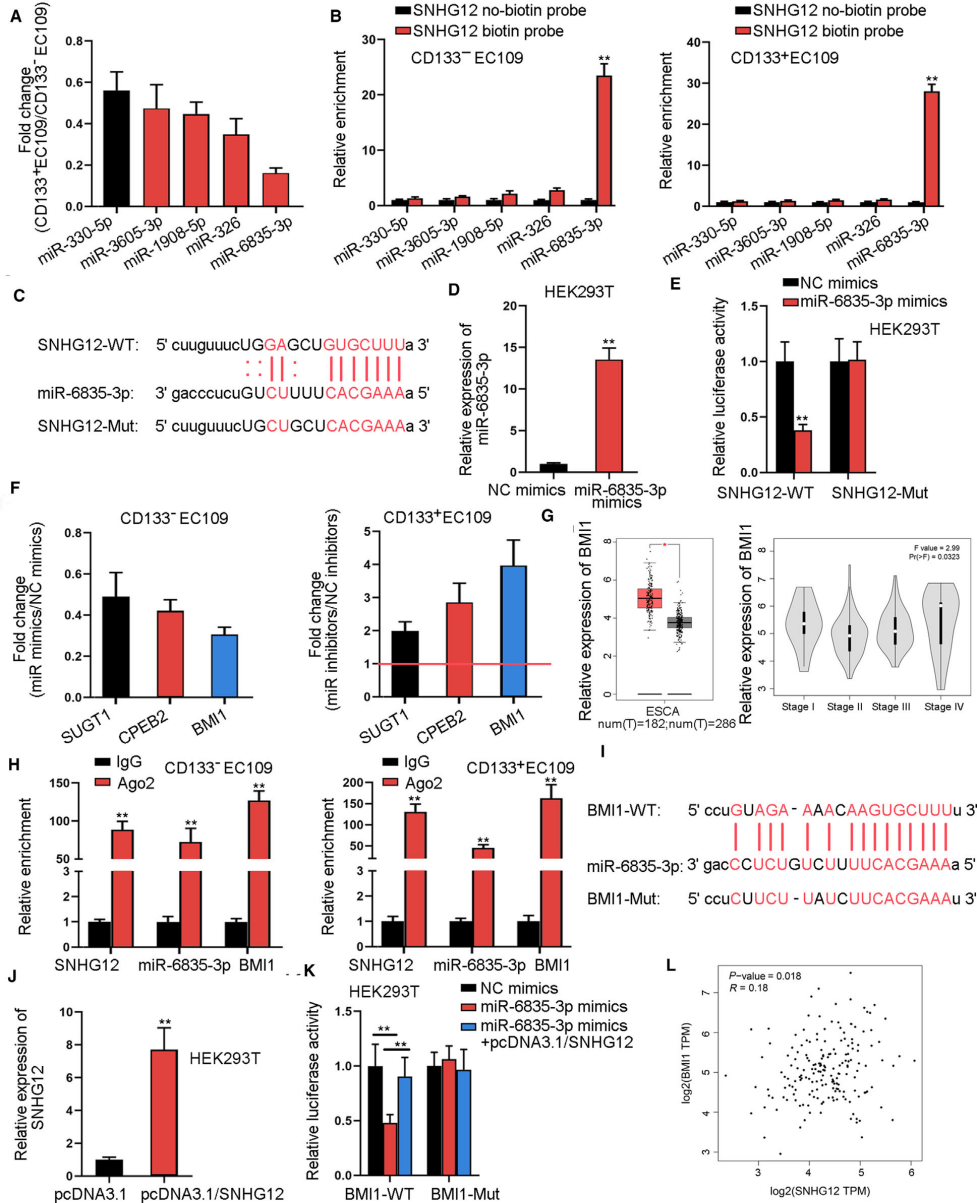
SNHG12 upregulates the expression of BMI1 via sequestering miR‐6835‐3p in ESCC. (A) qRT‐PCR data of fold change of several miRNA levels CD133^+^ESCC versus CD133^‐^ ESCC cells (*n* = 5; Student’s *t*‐test). (B) RNA pull‐down assay tested the relative enrichment of above‐mentioned miRNAs in the SNHG12 biotin probe group and the SNHG12 no‐biotin probe group (*n* = 5; Student’s *t*‐test). (C) starBase predicted the binding sites between SNHG12 and miR‐6835‐3p. (D) qRT‐PCR measured the overexpression efficiency of miR‐6835‐3p (*n* = 5; Student’s *t*‐test). (E) Luciferase reporter assay measured the luciferase activity of SNHG12‐WT and SNHG12‐Mut under miR‐6835‐3p overexpression (*n* = 4; Student’s *t*‐test). (F) qRT‐PCR data of the fold change of SUGT1, CPEB2, and BMI1 levels in CD133^‐^ ESCC cells with miR‐6835‐3p mimics or miR‐6835‐3p inhibitors versus NC mimics or NC inhibitors (*n* = 5; Student’s *t*‐test). (G) The expression of BMI1 in ESCA (esophageal carcinoma) tissues versus corresponding normal tissues and the expression of BMI1 in ESCA patients at different cancer stages were obtained from TCGA database. (H) RIP assay detected the relative enrichment of SNHG12, miR‐6835‐3p, and BMI1 in the Ago2 group (*n* = 5; Student’s *t*‐test). (I) starBase predicted the binding sites between BMI1 and miR‐6835‐3p. (J) qRT‐PCR measured the overexpression efficiency of SNHG12 (*n* = 5; Student’s *t*‐test). (K) Luciferase reporter assay measured the luciferase activity of BMI1‐WT and BMI1‐Mut (*n* = 4; one‐way ANOVA). (L) The positive correlation between SNHG12 expression and BMI1 expression was obtained from TCGA database. Results were all exhibited as the mean ± standard deviation (SD) and taken from more than three independent experiments. **P < 0.01

Thereafter, we aimed to explore the downstream regulation mechanism of SNHG12. Based on the previous observation of the cytoplasmic distribution of SNHG12, we deduced that SNHG12 regulated certain genes post‐transcriptionally. Since lncRNAs were extensively supported to participate in post‐transcriptional regulation through ceRNA mechanism, we screened out a cluster of microRNA (miRNAs) possibly bind with SNHG12 from starBase. Then, qRT‐PCR identified the top five miRNAs (miR‐330‐5p, miR‐3605‐3p, miR‐1908‐5p, miR‐326, and miR‐6835‐3p) with the lowest expression in ESCC cells, and miR‐6835‐3p exhibited the lowest expression among the 5 miRNAs (Fig. 4A). RNA pull‐down data supported that only miR‐6835‐3p was enriched in SNHG12 biotin probe but not SNHG12 no‐biotin probe group among above miRNAs (Fig. 4B). Further, the predicted miR‐6835‐3p sites in SNHG12 were obtained from starBase and were mutated to generate SNHG12‐Mut (Fig. 4C). Besides, the satisfying overexpression efficiency of miR‐6835‐3p was obtained by miR‐6835‐3p mimics in HEK 293T cells (Fig. 4D). Overexpressing miR‐6835‐3p inhibited the luciferase activity of SNHG12‐WT and failed to alter SNHG12‐Mut activity (Fig. 4E). Moreover, three messenger RNAs (mRNAs) (SUGT1, CPEB2, and BMI1) potently binding with miR‐6835‐3p were screened out by starBase. Data from qRT‐PCR demonstrated BMI1 expression was downregulated most significantly by miR‐6835‐3p mimics and upregulated most significantly by miR‐6835‐3p inhibitors (Fig. 4F). According to TCGA public database GEPIA, BMI1 level was upregulated in ESCA (esophageal carcinoma) tissues, and ESCA patients in terminal stage (IV) presented higher BMI1 level than those in earlier stages (I‐II) (Fig. 4G). RIP assay showed the obvious enrichment of SNHG12, miR‐6835‐3p, and BMI1 in the Ago2 group (Fig. 4H). Additionally, starBase was utilized to predict the binding sites between miR‐6835‐3p and BMI1 (Fig. 4I). Then, we verified that pcDNA3.1/SNHG12 increased SNHG12 expression in HEK 293T cells (Fig. 4J). Overexpressing miR‐6835‐3p suppressed BMI1‐WT luciferase activity, and this inhibitory impact was offset by SNHG12 overexpression, with the luciferase activity of BMI1‐Mut unaffected (Fig. 4K). Finally, the positive correlation between SNHG12 and BMI1 expressions in tissues was obtained by TCGA database (Fig. 4L). To sum up, SNHG12 upregulated BMI1 expression via sequestering miR‐6835‐3p in ESCC.

### 3.5 BMI1 is a target for SNHG12 to regulate the development of ESCC

**Fig. 5:**
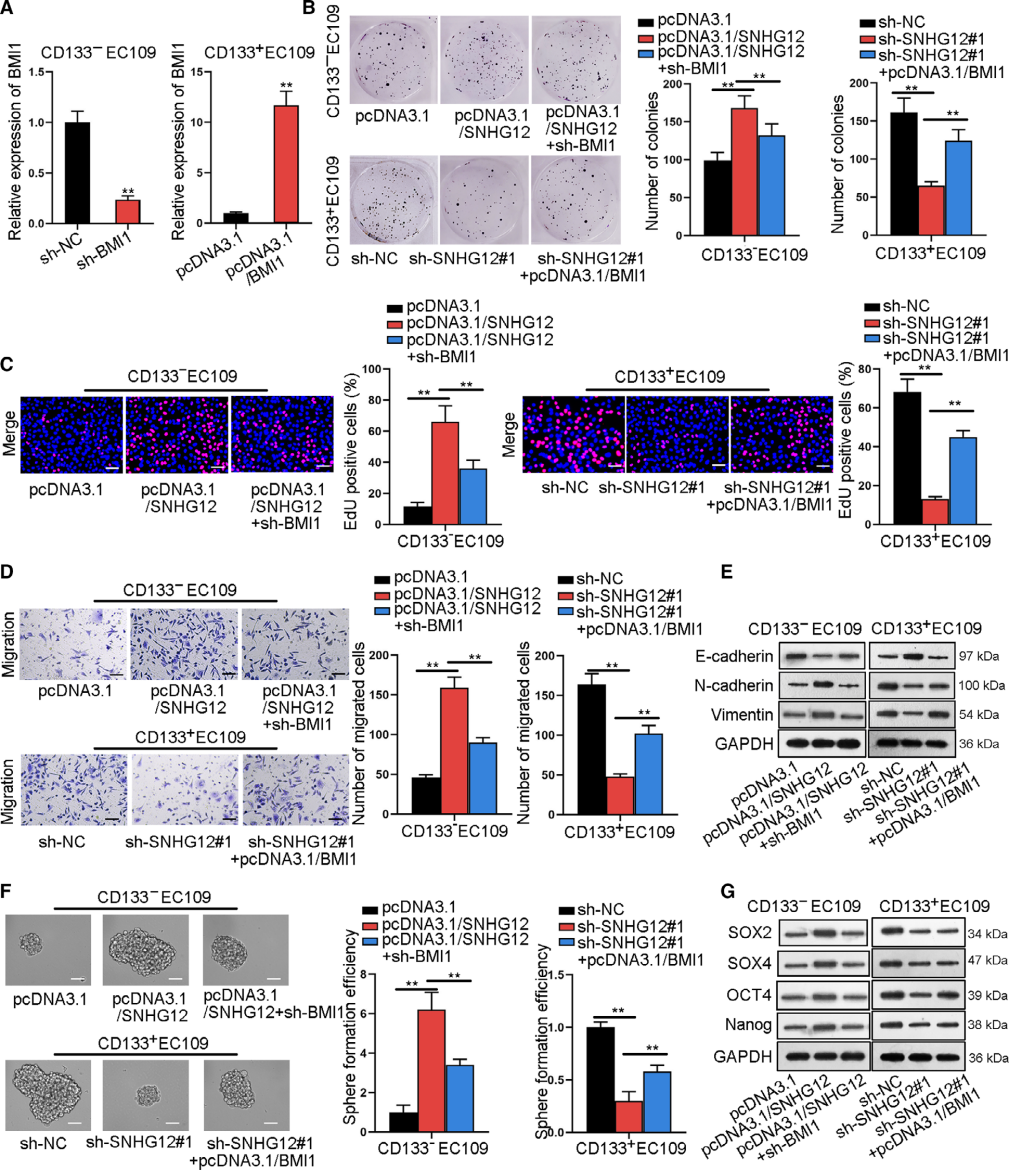
BMI1 is a target for SNHG12 to regulate ESCC development. (A) qRT‐PCR measured the knockdown and overexpression efficiency of BMI1 (*n* = 5; Student’s *t*‐test). (B,C) Colony formation and EdU (bar value = 100 μm) assays detected ESCC cell proliferation under different transfecting conditions (*n* = 5; one‐way ANOVA). (D) Transwell assay measured ESCC cell migration ability (bar value = 100 μm; *n* = 5; one‐way ANOVA). (E) Western blot assay tested the expression of EMT‐related protein (E‐cadherin, N‐cadherin, and vimentin; *n* = 5). (F) Sphere formation assay detected the ESCC cell sphere formation efficiency (bar value = 100 μm; *n* = 5; one‐way ANOVA). (G) Western blot assay measured the protein expression of stem cell markers (SOX2, SOX4, OCT4, and Nanog; *n* = 5). Results were all exhibited as the mean ± standard deviation (SD) and taken from more than three independent experiments. **P < 0.01

To detect whether SNHG12 regulated cellular activities in ESCC via BMI1, rescuing experiments were performed. Firstly, we verified that BMI1 expression was remarkably downregulated or upregulated by sh‐BMI1 or pcDNA3.1/BMI1 in ESCC cells (Fig. 5A). Afterward, we observed that sh‐BMI1 partially alleviated the stimulating effect of pcDNA3.1/SNHG12 on cell proliferation, and pcDNA3.1/BMI1 partially reversed the repressive impact of sh‐SNHG12#1 on cell proliferation (Fig. 5B,C). Besides, overexpression of BMI1 partly restored cell migration and invasion that was repressed by sh‐SNHG12#1, and silence of BMI1 partly abrogates migration and invasion encouraged by pcDNA3.1/SNHG12 (Figs 5D and S5A). Moreover, upregulation of E‐cadherin and decline of N‐cadherin and vimentin caused by pcDNA3.1/SNHG12 were partially rescued by BMI1 deficiency. In the meantime, BMI1 overexpression partially reversed the decline of E‐cadherin and upregulation of N‐cadherin and vimentin caused by sh‐SNHG12#1 (Fig. 5E). Moreover, BMI1 silence partly counteracted the encouraging effect of pcDNA3.1/SNHG12 on sphere formation and expression of stemness markers (SOX2, SOX4, OCT4, and Nanog), and BMI1 overexpression partly rescued sphere formation and expression of stemness genes that were repressed by SNHG12 knockdown (Fig. 5F,G). Knockdown of BMI1 partly reversed the increase of CD133^+^ ratio in ESCC cells with SNHG12 overexpression, and overexpressing BMI1 partly restored CD133^+^ ratio in ESCC cells with SNHG12 knockdown (Fig. S5B). Overall, BMI1 was a target for SNHG12 to regulate the development of ESCC, and the partial rescue presented by these data indicated that SNHG12 might have alternate targets in ESCC.

### 3.6 SNHG12 enhances the mRNA stability of CTNNB1 via recruiting IGF2BP2 in ESCC

**Fig. 6:**
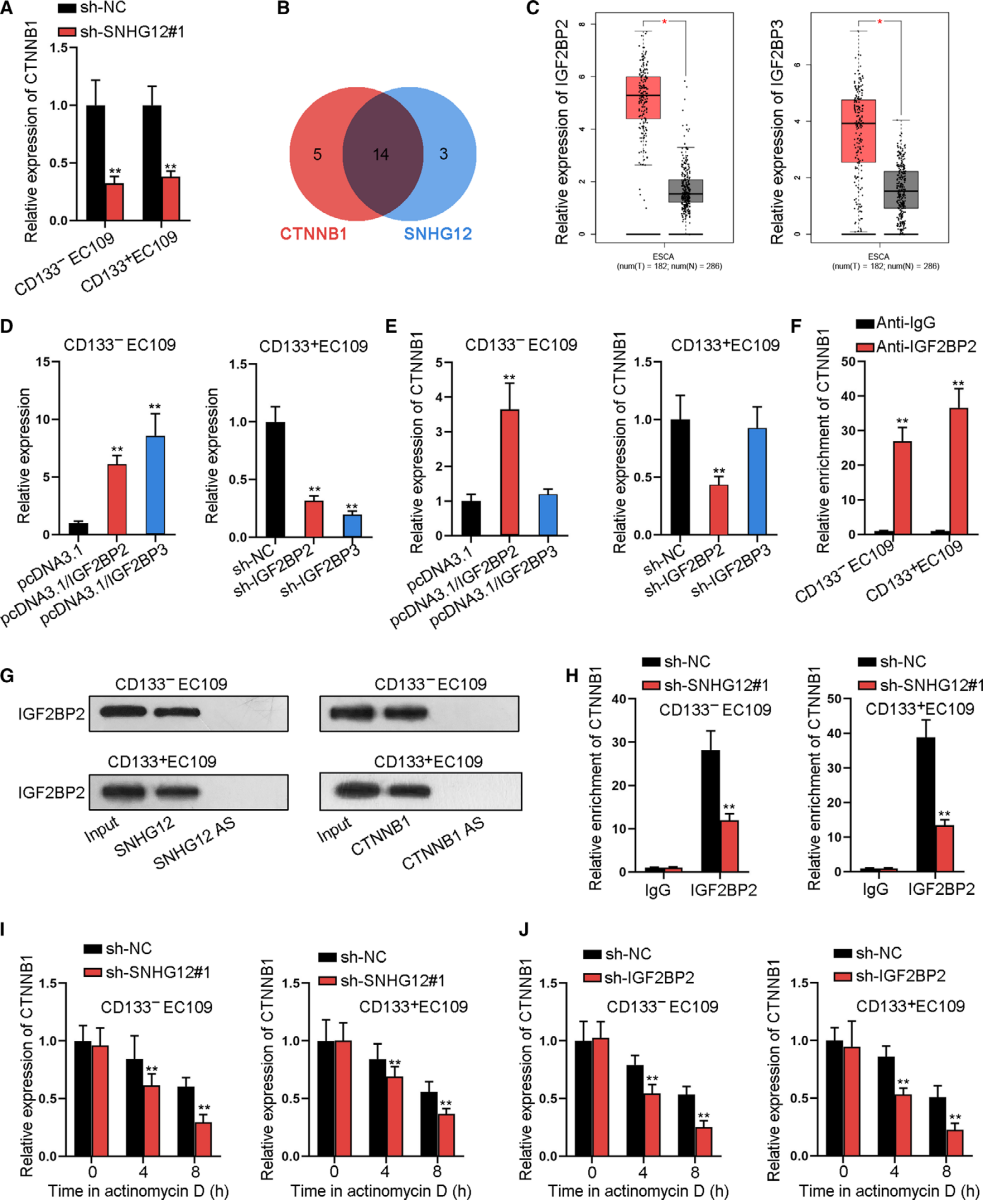
SNHG12 enhances the mRNA stability of CTNNB1 via recruiting IGF2BP2 in ESCC. (A) qRT‐PCR measured the expression of CTNNB1 when silencing SNHG12 (*n* = 5; Student’s *t*‐test). (B) Fourteen RBPs interacting with CTNNB1 and SNHG12 were screened out through starBase. (C) The expression of IGF2BP2 and IGF2BP3 in ESCA (esophageal carcinoma) tissues and corresponding normal tissues was obtained from TCGA database. (D) qRT‐PCR measured the overexpression and knockdown efficiency of IGF2BP2 and IGF2BP3 (*n* = 5; one‐way ANOVA). (E) qRT‐PCR measured the expression of CTNNB1 under IGF2BP2/3 overexpression or knockdown (*n* = 5; one‐way ANOVA). (F) RIP assay detected the relative enrichment of CTNNB1 in the anti‐IGF2BP2 group (*n* = 5; Student’s *t*‐test). (G) RNA pull‐down–western blot assay measured IGF2BP2 enrichment in the SNHG12 or SNHG12 antisense group (*n* = 5). (H) RIP assay detected the relative enrichment of CTNNB1 in the anti‐IGF2BP2 group when knocking down SNHG12 (n = 5; Student’s *t*‐test). (I,J) qRT‐PCR measured CTNNB1 expression after actinomycin D treatment in different transfecting conditions (*n* = 5; Student’s *t*‐test). Results were all exhibited as the mean ± standard deviation (SD) and taken from more than three independent experiments. *P < 0.05, **P < 0.01

It was reported that BMI1 can activate Wnt pathway (Li *et al.*, 2018a; Yu *et al.*, 2018), and Wnt/β‐catenin pathway was extensively supported as a contributing signaling for cancer cell growth, stemness, and EMT (Fodde and Brabletz, 2007; Reya and Clevers, 2005). Therefore, we speculated that SNHG12 modulated Wnt/β‐catenin pathway through BMI1. Interestingly, we found that sh‐SNHG12#1 reduced Wnt‐associated genes (β‐catenin, SOX2, SOX4, C‐myc, and MMP7), and such effect was partly reversed by overexpressing BMI1. Overexpressing SNHG12 increased the Wnt‐related proteins, and such effect was partly counteracted by BMI1 knockdown (Fig. S6A). Hence, we deduced that the SNHG12 had alternate target genes to regulate Wnt signaling. As axiomatically known, β‐catenin, encoded by CTNNB1, was the main factor in canonical Wnt signaling. Data of qRT‐PCR revealed that SNHG12 depletion decreased CTNNB1 expression (Fig. 6A). Furthermore, 14 common RBPs (RNA‐binding proteins) binding to SNHG12 and CTNNB1 were predicted by starBase (Fig. 6B). Based on TCGA database, we discovered that expressions of IGF2BP2 and IGF2BP3 were significantly upregulated in ESCA tissues compared with normal tissues (Fig. 6C), and the expressions of the other 12 RBPs were not altered in tumor tissues versus normal tissues (Fig. S7A–L). Later on, the knockdown and overexpression efficiency of IGF2BP2/IGF2BP3 were verified (Fig. 6D). The expression of CTNNB1 was upregulated or downregulated by pcDNA3.1/IGF2BP2 or sh‐IGF2BP2, but was not affected by pcDNA3.1/IGF2BP3 or sh‐IGF2BP3 (Fig. 6E). Besides, RIP assay showed the high enrichment of CTNNB1 in anti‐IGF2BP2 precipitates (Fig. 6F). RNA pull‐down and western blot assay also verified that the pulled down products of the SNHG12/CTNNB1 (sense) group showed obvious IGF2BP2 enrichment (Fig. 6G). Additionally, RIP assay confirmed that the enrichment of CTNNB1 in the anti‐IGF2BP2 group was decreased when knocking down SNHG12 (Fig. 6H). Finally, after actinomycin D treatment, qRT‐PCR showed that silencing SNHG12 or IGF2BP2 accelerated the degradation of CTNNB1 (Fig. 6I,J). In a summary, SNHG12 could enhance the mRNA stability of CTNNB1 via recruiting IGF2BP2 in ESCC.

### 3.7 CTNNB1 and BMI1 mediate the regulatory function of SNHG12 in ESCC

**Fig. 7:**
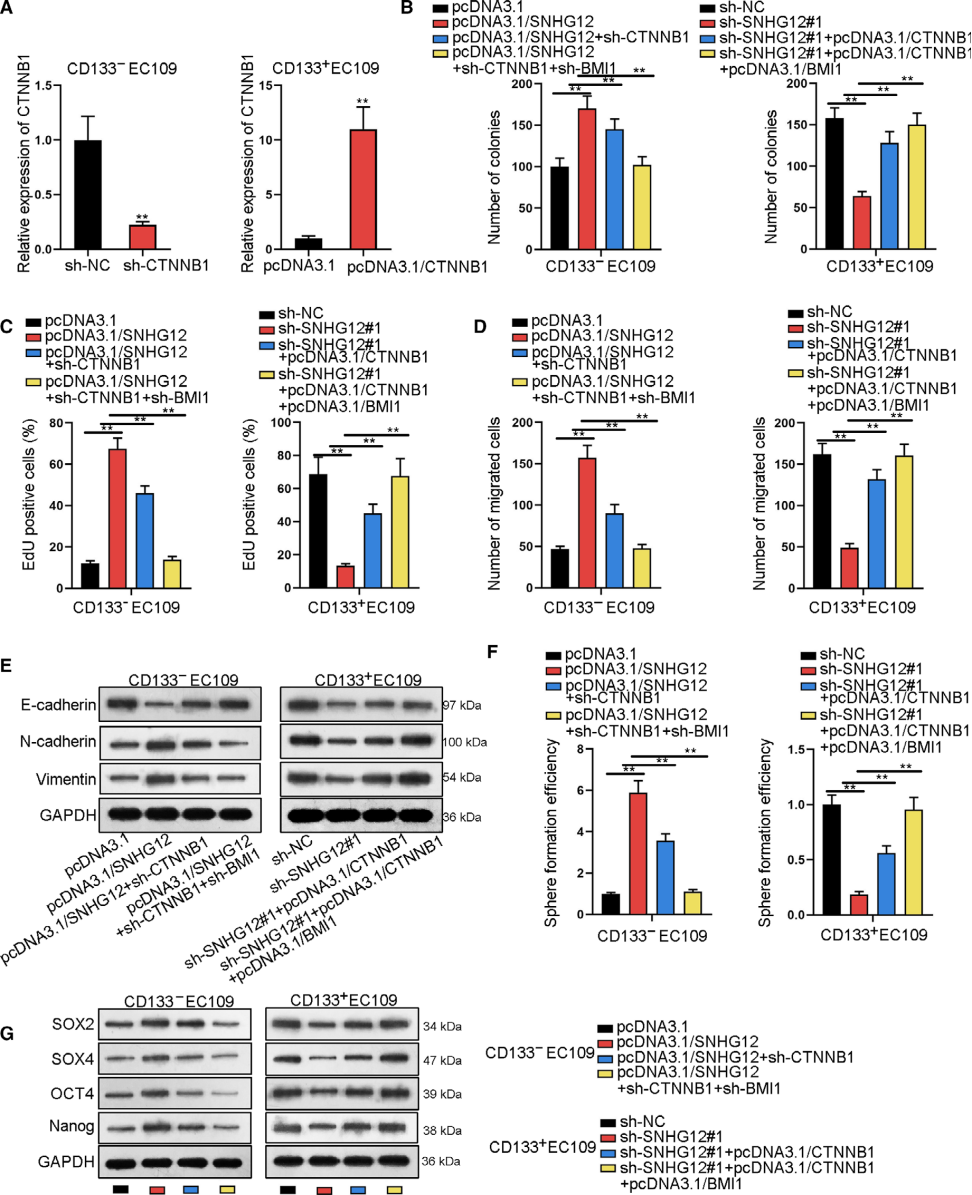
CTNNB1 and BMI1 mediate the regulation of SNHG12 in ESCC. (A) qRT‐PCR measured the knockdown and overexpression efficiency of CTNNB1 (*n* = 5; Student’s *t*‐test). (B,C) Quantification of colony numbers and EdU‐positive cell ratio reflected ESCC cell proliferation under different transfecting conditions (*n* = 5; one‐way ANOVA). (D) Transwell assay measured ESCC cell migration ability (*n* = 5; one‐way ANOVA). (E) Western blot assay tested the expression of EMT‐related proteins (E‐cadherin, N‐cadherin, and vimentin; *n* = 5). (F) Sphere formation efficiency of ESCC cells with indicated transfections (*n* = 5; one‐way ANOVA). (G) Western blot assay measured the protein expression of stem cell markers (SOX2, SOX4, OCT4, and Nanog; *n* = 5). Results were all exhibited as the mean ± standard deviation (SD) and taken from more than three independent experiments. **P < 0.01

Later, we determined whether SNHG12 functioned in ESCC through CTNNB1 and BMI1. Firstly, CTNNB1 expression was downregulated or upregulated by sh‐CTNNB1 or pcDNA3.1/CTNNB1 (Fig. 7A). Consequently, sh‐CTNNB1 partly abrogated the proliferation promoted by pcDNA3.1/SNHG12, and co‐overexpression of CTNNB1 and BMI1 further strengthened such counteraction (Fig. 7B,C). CTNNB1 overexpression partly restored proliferation repressed by sh‐SNHG12#1, and joint overexpression of CTNNB1 and BMI1 furthered the restoration (Fig. 7B,C). Sh‐CTNNB1 impaired ESCC cell migration, invasion, and EMT process that was facilitated by pcDNA3.1/SNHG12, and such impairment was strengthened by jointly knocking down CTNNB1 and BMI1. CTNNB1 overexpression counteracted repressing effect of sh‐SNHG12#1 on migration, invasion, and EMT in ESCC cells, and such counteraction was furthered by co‐overexpression of CTNNB1 and BMI1 (Figs 7D,E and S8A). Transfecting sh‐CTNNB1 impaired sphere formation that was facilitated by pcDNA3.1/SNHG12 in ESCC cells, and cotransfection of sh‐BMI1 strengthened such impairment. Sphere formation repressed by sh‐SNHG12#1 was restored by CTNNB1 overexpression, and such restoration was enhanced by cotransfection of pcDNA3.1/BMI1 (Fig. 7F,G). CD133 + ratio increased by SNHG12 overexpression was partly reversed by CTNNB1 knockdown and was fully reversed by the knockdown of CTNNB1 and BMI1. The effect of SNHG12 knockdown on reducing CD133 + ratio was partly counteracted by CTNNB1 overexpression and was fully counteracted by co‐overexpression of CTNNB1 and BMI1 (Fig. S8B). Altogether, CTNNB1 and BMI1 mediated the regulatory function of SNHG12 in ESCC.

### 3.8 SOX4 binds with SNHG12 promoter to transcriptionally activate SNHG12 in ESCC

**Fig. 8:**
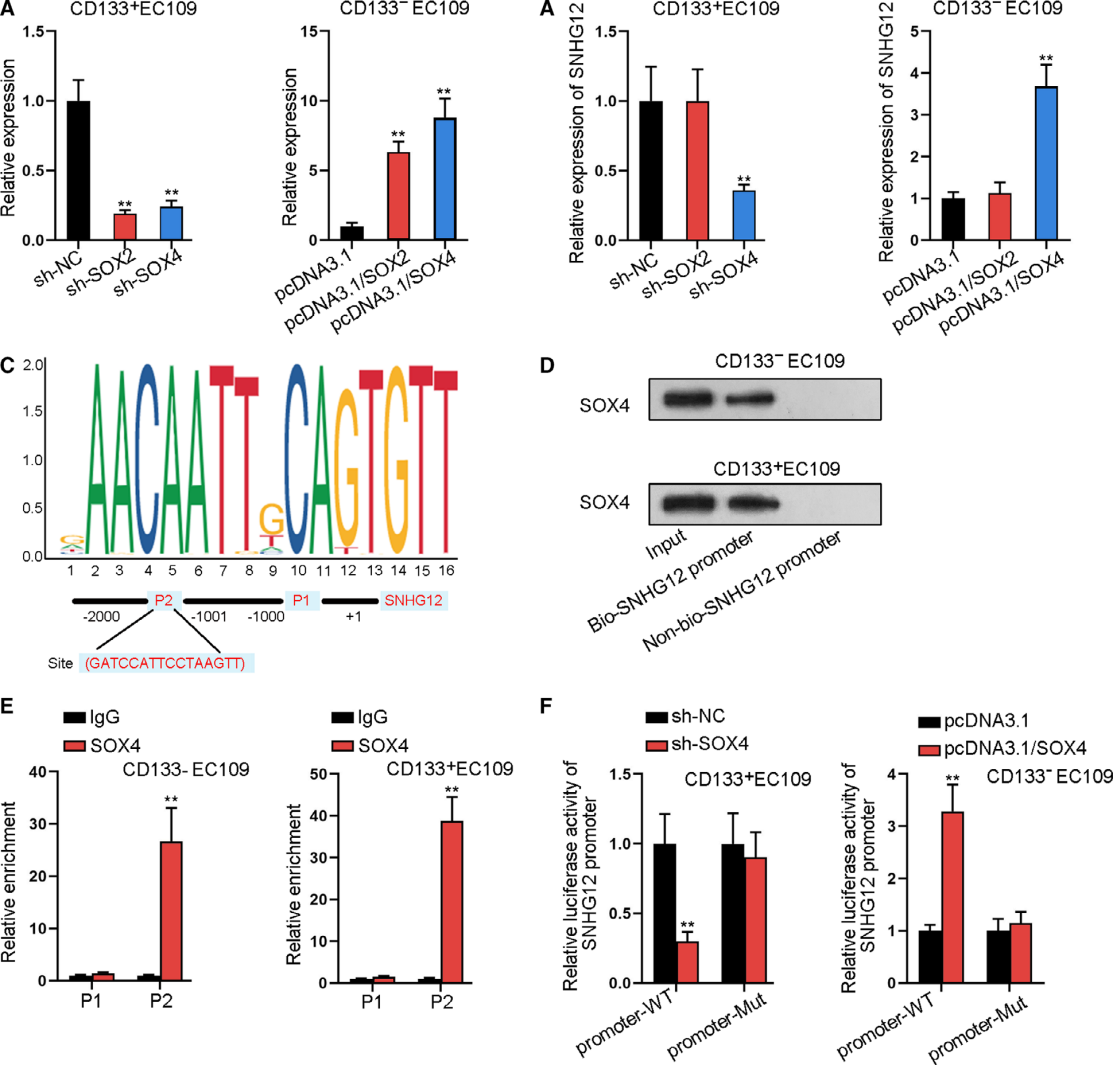
SOX4 binds with SNHG12 promoter to transcriptionally activate SNHG12 in ESCC. (A) qRT‐PCR measured the knockdown and overexpression efficiency of SOX2/SOX4 (n = 5; one‐way ANOVA). (B) qRT‐PCR measured SNHG12 expression when knocking down or overexpressing SOX2/SOX4 (*n* = 5; one‐way ANOVA). (C) JASPAR and UCSC websites were utilized to obtain the motif of SOX4 as well as the binding site of SOX4 in SNHG12 promoter. (D) DNA pull‐down–western blot assay detected the enrichment of SOX4 in SNHG12 promoter pull‐down (*n* = 5). (E) ChIP assay tested the relative enrichment of SNHG12 promoter fractions (P1 without predicted site and P2 with predicted site) in SOX4 ChIP products (*n* = 5; Student’s *t*‐test). (F) Luciferase reporter assay measured the luciferase activity of SNHG12 promoter‐WT and SNHG12 promoter‐Mut (n = 5; Student’s *t*‐test). Results were all exhibited as the mean ± standard deviation (SD) and taken from more than three independent experiments. **P < 0.01

Moreover, the effects of Wnt pathway downstream mRNAs on SNHG12 promoter were confirmed. Among the key downstream genes, we found that SOX2 or SOX4 expression changes could influence the expression of SNHG12. First of all, the knockdown and overexpression efficiency of SOX2/SOX4 were measured. Sh‐SOX2 and sh‐SOX4 declined, and pcDNA3.1/SOX2 or pcDNA3.1/SOX4 increased (Fig. 8A). Subsequently, qRT‐PCR showed that SNHG12 expression was downregulated by sh‐SOX4 and upregulated by pcDNA3.1/SOX4, but was not affected by sh‐SOX2 or pcDNA3.1/SOX2 (Fig. 8B). Through JASPAR and UCSC websites, the motif of SOX4 and the binding site of SOX4 in SNHG12 promoter were obtained (Fig. 8C). Further, DNA pull‐down–western blot assay revealed the obvious enrichment of SOX4 in the bio‐SNHG12 promoter group rather than the non‐bio‐SNHG12 promoter group (Fig. 8D). Chromatin immunoprecipitation (ChIP) assay confirmed the high enrichment of SNHG12 promoter in the anti‐SOX4 group (P2 area) (Fig. 8E). Lastly, luciferase activity of SNHG12 promoter was inhibited or stimulated by sh‐SOX4 or pcDNA3.1/SOX4 (Fig. 8F). Thus, SOX4 binds with SNHG12 promoter to transcriptionally activate SNHG12 in ESCC.

### 3.9 SNHG12 knockdown retards tumorigenesis and reduces metastasis in ESCC *in vivo*

Finally, we determined whether SNHG12 functioned in ESCC via establishing animal model. To monitor tumorigenesis, CD133^+^ EC109 cells transfected with sh‐NC or sh‐SNHG12#1 were subcutaneously inoculated into mice to generate xenografts. As observed, growth of tumors in mice was slower in the sh‐SNHG12#1 group than in the sh‐NC group (Fig. S9A). At day 28, the weight of resected tumors from mice in the sh‐SNHG13#1 group was lighter than that in the sh‐NC group (Fig. S9B). qRT‐PCR results validated that levels of SNHG12, BMI1, and CTNNB1 were lower in xenografts with SNHG12 knockdown than control (Fig. S9C). Besides, the protein levels of BMI1, β‐catenin, the downstream factors c‐Myc and MMP7, and EMT‐related N‐cadherin and vimentin were reduced but level of E‐cadherin was increased in xenografts with sh‐SNHG12#1 (Fig. S9D). Stemness‐specific proteins including SOX2, SOX4, OCT4, and Nanog were lessened under SNHG12 knockdown *in vivo* (Fig. S9E). Through IHC staining, we discovered that proliferation indexes including Ki‐67 and PCNA declined in xenografts with SNHG12 knockdown (Fig. S9F). Additionally, mice were subjected to tail‐vein injection of CD133^+^ EC109 cells with sh‐NC or sh‐SNHG12#1 to establish metastasis model. Consequently, sh‐SNHG12#1 led to less metastasis nodules in mice (Fig. S9G). Together, SNHG12 knockdown retards tumorigenesis and reduces metastasis in ESCC *in vivo*.

## 4 Discussion

Abundant evidences illustrate that lncRNAs usually exhibit aberrant expression in various tumors and serve as a vital modulator of biological process, including cell proliferation, migration, and stemness as well as EMT process. For illustration, lncRNA MEG3 suppresses cell proliferation and metastasis in gastric cancer through p53 pathway (Wei and Wang, 2017). LncRNA UCA1 facilitates the migration and invasion of pancreatic cancer cells through Hippo signaling pathway (Zhang *et al.*, 2018). LncRNA PTAR accelerates epithelial**–**mesenchymal transition and cell invasion–metastasis in serous ovarian cancer via sponging with miR‐101‐3p to modulate the expression of ZEB1 (Liang *et al.*, 2018a). LncRNA MALAT1 strengthens cell stemness in gastric cancer by enhancing the mRNA stability of SOX2 (Xiao *et al.*, 2019). Similarly, in ESCC, the participation of lncRNAs has been documented. For instance, lncRNA CASC9 contributes to ESCC development via regulating PDCD4 expression through EZH2 (Wu *et al.*, 2017). LncRNA CASC9 motivates ESCC metastasis by upregulating LAMC2 expression through the interaction with the CREB‐binding protein (Liang *et al.*, 2018b). Linc‐ROR expedites ESCC progression via the inhibition of SOX9 (Wang *et al.*, 2017a). In our research, we discovered that 3 lncRNAs from SNHG family (SNHG1, SNHG7, and SNHG12) were upregulated in ESCA (esophageal carcinoma) tissues based on Cancer RNA‐Seq Nexus analysis. Previously, the 3 lncRNAs have been extensively reported to contribute to carcinogenesis (Li *et al.*, 2018b; Lin *et al.*, 2017; Wang *et al.*, 2019b; Wang *et al.*, 2017b; Wang *et al.*, 2018; Xu *et al.*, 2018b). Among them, SNHG1 and SNHG7 have been related to ESCC by former studies (Xu *et al.*, 2018a; Yan *et al.*, 2017), but SNHG12 was first linked to ESCC in our study. We confirmed that SNHG12 expression was upregulated in CD133^+^ ESCC cells compared with CD133^‐^ cancer cells, and we also validated that SNHG12 was mainly distributed in cytoplasm. Functional assay illustrated that SNHG12 upregulation promoted cell proliferation, migration, EMT, and stemness in CD133^‐^ESCC cells, and SNHG12 depletion brought about on the contrary results in CD133^+^ cells, suggesting the oncogenic role of SNHG12 in ESCC.

The LncRNAs usually serve as the sponge of microRNAs (miRNAs) to modulate the downstream target gene expression in cancers (Zhao *et al.*, 2017). This study first discovered that SNHG12 could interact with miR‐6835‐3p which was significantly lowly expressed in ESCC cells. MiRNAs affect gene expression by binding to the 3′ UTRs of target mRNAs (Ambros, 2004; Bartel, 2004). Here, among a group of predicted mRNAs, BMI1 was found to be the most significantly affected under miR‐6835‐3p overexpression or knockdown in ESCC cells. According to TCGA database, BMI1 expression was upregulated in ESCA tissues and was highly expressed in ESCA patients in terminal stage (IV), indicating its involvement in ESCC. Further, we first showed that SNHG12 could modulate BMI1 expression via sponging miR‐6835‐3p in ESCC cells. Besides, analysis based on TCGA database indicated that SNHG12 expression was positively correlated with BMI1 expression in ESCA tissues.

The effects of Wnt pathway in cancer development are extensively supported (Feng *et al.*, 2018; Gao *et al.*, 2018). Notably, Wnt/β‐catenin signaling is widely reported to regulate EMT and cell stemness in cancers by transcriptionally activating EMT and stemness‐related genes (Fodde and Brabletz, 2007; Reya and Clevers, 2005). For instance, lncRNA DANCR induced CTNNB1 to enhance the stemness of hepatocellular cancer cells and drive tumorigenesis (Yuan *et al.*, 2016). FOXP1 activates Wnt/β‐catenin in lung cancer to promote EMT (Yang *et al.*, 2017). In ESCC, lncRNA TUG1 increases EMT markers through Wnt/β‐catenin (Tang *et al.*, 2020). MiR‐942 enhances stem cell‐like characteristics in ESCC cells via Wnt/β‐catenin activation (Ge *et al.*, 2015). Members of SNHG family, including SNHG12, are supported to promote cancer development via regulating Wnt/β‐catenin pathway (Feng *et al.*, 2018; Shao *et al.*, 2019; Song *et al.*, 2019). As previously reported, BMI1 upregulation could activate Wnt pathway (Li *et al.*, 2018a; Yu *et al.*, 2018). Therefore, it is reasonable to suggest that SNHG12 contributed to stemness and EMT in ESCC cells through BMI/Wnt/β‐catenin pathway.

Interestingly, we elucidated that pcDNA3.1/BMI1 or sh‐BMI1 only partially offset the suppressing or stimulating influence of SNHG12 depletion or upregulation in Wnt pathway. Therefore, we further explored whether SNHG12 regulated Wnt pathway‐associated protein expression via post‐transcriptional mechanism in ESCC progression. We found that RBP (RNA‐binding protein) IGF2BP2 could cooperate with SNHG12 to enhance the mRNA stability of CTNNB1. Further, we suggested through rescue assay that BMI1 and CTNNB1 were two targets for SNHG12 to regulate ESCC progression. Subsequently, among the Wnt pathway downstream target genes, the transcription factor role of SOX4 in regulating expression of noncoding RNAs (ncRNAs) has been widely researched (Ding *et al.*, 2019). Our data first supported that SOX4 could transcriptionally activate SNHG12 and bind with SNHG12 promoter in ESCC. Finally, we provided *in vivo* data to prove that SNHG12 drove tumorigenesis and metastasis in ESCC.

## 5 Conclusion

This study first discovered that lncRNA SNHG12 induced proliferation, migration, EMT, and stemness of ESCC cells *in vitro* and drives tumorigenesis and metastasis *in vivo*. Mechanistically, SNHG12 sequesters miR‐6835‐3p to induce BMI1 and interacted with IGF2BP2 to stabilize CTNNB1 and activated Wnt/β‐catenin pathway (Fig. S10). These findings provided innovative thoughts for advancing the treatment of ESCC.

## Authors’ contribution

DW and XH codesigned this study and performed experiments. WW, XH, KW, and MW contributed to data, methods, and investigation. All authors approved final manuscript.

## Conflicts of interest

The authors declare no conflict of interest.

## Supporting information


**Fig. S1.** SNHG12 level in ESCC specimens and its prognostic value. (A) qRT‐PCR of SNHG12 level in ESCC specimens versus paired para‐tumor tissues (n = 5; Paired student’s t‐test). (B) Kaplan–Meier analysis of correlation between SNHG12 level and overall survival in ESCC patients (log‐rank test). Results were all exhibited as the mean ± Standard Deviation (SD) and taken from more than three independent experiments. **P < 0.01.

**Fig. S2.** Sorting and enriching of CD133^+^ ESCC cells. (A) CD133^+^ ESCC cells were sorted by flow cytometry analysis. (B) Sphere formation was used to enrich CD133^+^ ESCC cells, and CD133^+^ ratio in tumorspheres derived by ESCC cells versus the parental cells was analyzed by flow cytometry (n = 5; Student’s t‐test). (C) Western blot of stemness specific genes in tumorspheres derived by ESCC cells versus the parental cells (n = 5). Results were all exhibited as the mean ± Standard Deviation (SD) and taken from more than three independent experiments. **P < 0.01.

**Fig. S3.** Effect of SNHG12 overexpression on cell invasion and CD133^+^ ratio. (A) Pictures (bar value = 100 μm) of invasive ESCC cells in transwell system under SNHG12 overexpression and the number of cells per field was quantified (n = 5; Student’s *t*‐test). (B) CD133^+^ ratio in ESCC cells with SNHG12 overexpression was quantified by flow cytometry analysis (n = 5; Student’s t‐test). (C) Original data of western blot in Figure 2G (n = 5). Results were all exhibited as the mean ± Standard Deviation (SD) and taken from more than three independent experiments. **P < 0.01.

**Fig. S4.** Effects of SNHG12 knockdown on cell invasion and CD133^+^ ratio. (A) Pictures (bar value = 100 μm) of invasive ESCC cells in transwell system under SNHG12 knockdown and the number of cells per field was quantified (n = 5; one‐way ANOVA). (B) CD133^+^ ratio in ESCC cells with SNHG12 knockdown was quantified by flow cytometry analysis (n = 5; one‐way ANOVA). Results were all exhibited as the mean ± Standard Deviation (SD) and taken from more than three independent experiments. **P < 0.01.

**Fig. S5.** BMI1 rescued SNHG12 function in cell invasion and CD133^+^ ratio in ESCC. (A) Pictures (bar value = 100 μm) of invasive ESCC cells in transwell system with indicated transfections and the number of cells per field was quantified (n = 5; one‐way ANOVA). (B) CD133^+^ ratio in ESCC cells with indicated transfections was quantified by flow cytometry analysis (n = 5; one‐way ANOVA). Results were all exhibited as the mean ± Standard Deviation (SD) and taken from more than three independent experiments. **P < 0.01.

**Fig. S6.** Western blot of key factors in Wnt pathway. (A) Western blot assay measured the Wnt pathway‐related protein level in different transfected groups (n = 5). Results were all exhibited as the mean ± Standard Deviation (SD) and taken from more than three independent experiments.

**Fig. S7.** Analysis of RBP expressions in ESCA samples from GEPIA. (A‐L) The expression of the expression of other twelve RBPs in ESCA (esophageal carcinoma) tissues and corresponding normal tissues was obtained from TCGA database. Results were all exhibited as the mean ± Standard Deviation (SD) and taken from more than three independent experiments.

**Fig. S8.** BMI1 and CTNNB1 rescue SNHG12 function in cell invasion and CD133^+^ ratio in ESCC. (A) Number of invasive ESCC cells in transwell system with indicated transfections per field was quantified (n = 5; one‐way ANOVA). (B) CD133^+^ ratio in ESCC cells with indicated transfections was quantified by flow cytometry analysis (n = 5; one‐way ANOVA). Results were all exhibited as the mean ± Standard Deviation (SD) and taken from more than three independent experiments. **P < 0.01.

**Fig. S9.** Function of SNHG12 *in vivo*. (A) Growth curve of xenografts in mice subcutaneously injected with CD133^+^ EC109 cells with sh‐NC or sh‐SNHG12#1 (n = 5; Student’s *t*‐test). (B) Tumor weight at day 28 after injection was detected (n = 5; Student’s *t*‐test). (C) qRT‐PCR of SNHG12, BMI1, and CTNNB1 levels in xenografts of each group (n = 5; Student’s t‐test). (D‐E) Western blots of BMI1, β‐catenin, c‐Myc, MMP7, E‐cadherin, N‐cadherin, Vimentin, SOX2, SOX4, OCT4, and Nanog in xenografts of each group (n = 5). (F) IHC picture of Ki‐67 and PCNA in xenografts of each group. Scale bar: 100 μm (n = 5; Student’s *t*‐test). (G) Pictures and quantification of HE staining of metastatic nodules in mice intravenously injected with CD133^+^ EC109 cells with sh‐NC or sh‐SNHG12#1. (Scale bar: 100 μm; n = 5; Student’s *t*‐test). Results were all exhibited as the mean ± Standard Deviation (SD) and taken from more than three independent experiments. **P < 0.01.

**Fig. S10.** In esophageal squamous cell carcinoma, SNHG12 regulates BMI1 expression via sponging miR‐6835‐3p, and enhances CTNNB1 stability via recruiting IGF2BP2. Thus, SNHG12 activates Wnt/β‐catenin signaling and facilitates proliferation, metastasis and stemness. Downstream of the Wnt pathway, SOX4 binds with SNHG12 promoter to transcriptionally activate SNHG12.

## References

[mol212683-bib-0001] Ambros V (2004) The functions of animal microRNAs. Nature 431, 350–355. 15372042 10.1038/nature02871

[mol212683-bib-0002] Bartel DP (2004) MicroRNAs: genomics, biogenesis, mechanism, and function. Cell 116, 281–297. 14744438 10.1016/s0092-8674(04)00045-5

[mol212683-bib-0003] Cai C , Huo Q , Wang X , Chen B and Yang Q (2017) SNHG16 contributes to breast cancer cell migration by competitively binding miR‐98 with E2F5. Biochem Biophys Res Comm 485, 272–278. 28232182 10.1016/j.bbrc.2017.02.094

[mol212683-bib-0004] Cheng G , Song Z , Liu Y , Xiao H , Ruan H , Cao Q , Wang K , Xiao W , Xiong Z , Liu D *et al* (2019) Long noncoding RNA SNHG12 indicates the prognosis of prostate cancer and accelerates tumorigenesis via sponging miR‐133b. J Cell Physiol 235, 1235–1246. 31267540 10.1002/jcp.29039

[mol212683-bib-0005] Ding L , Zhao Y , Dang S , Wang Y , Li X , Yu X , Li Z , Wei J , Liu M and Li G (2019) Circular RNA circ‐DONSON facilitates gastric cancer growth and invasion via NURF complex dependent activation of transcription factor SOX4. Mol Cancer 18, 45. 30922402 10.1186/s12943-019-1006-2 PMC6437893

[mol212683-bib-0006] Ding S , Qu W , Jiao Y , Zhang J , Zhang C and Dang S (2018) LncRNA SNHG12 promotes the proliferation and metastasis of papillary thyroid carcinoma cells through regulating wnt/beta‐catenin signaling pathway. Cancer Biomark 22, 217–226. 29630517 10.3233/CBM-170777 PMC13078429

[mol212683-bib-0007] Dong J , Teng F , Guo W , Yang J , Ding G and Fu Z (2018) lncRNA SNHG8 Promotes the Tumorigenesis and Metastasis by Sponging miR‐149‐5p and Predicts Tumor Recurrence in Hepatocellular Carcinoma. Cell Physiol Biochem 51, 2262–2274. 30537734 10.1159/000495871

[mol212683-bib-0008] Feng F , Chen A , Huang J , Xia Q , Chen Y and Jin X (2018) Long noncoding RNA SNHG16 contributes to the development of bladder cancer via regulating miR‐98/STAT3/Wnt/beta‐catenin pathway axis. J Cell Biochem 119, 9408–9418. 30132983 10.1002/jcb.27257

[mol212683-bib-0009] Fodde R and Brabletz T (2007) Wnt/β‐catenin signaling in cancer stemness and malignant behavior. Curr Opin Cell Biol 19, 150–158. 17306971 10.1016/j.ceb.2007.02.007

[mol212683-bib-0010] Gao J , Zhao C , Liu Q , Hou X , Li S , Xing X , Yang C and Luo Y (2018) Cyclin G2 suppresses Wnt/beta‐catenin signaling and inhibits gastric cancer cell growth and migration through Dapper1. J Exp Clin Cancer Res 37, 317. 30547803 10.1186/s13046-018-0973-2 PMC6295076

[mol212683-bib-0011] Ge C , Wu S , Wang W , Liu Z , Zhang J , Wang Z , Li R , Zhang Z , Li Z , Dong S *et al* (2015) miR‐942 promotes cancer stem cell‐like traits in esophageal squamous cell carcinoma through activation of Wnt/β‐catenin signalling pathway. Oncotarget 6, 10964–10977. 25844602 10.18632/oncotarget.3696 PMC4484432

[mol212683-bib-0012] Gibb EA , Brown CJ and Lam WL (2011) The functional role of long non‐coding RNA in human carcinomas. Mol Cancer 10, 38. 21489289 10.1186/1476-4598-10-38 PMC3098824

[mol212683-bib-0013] Hao Y , Wu W , Shi F , Dalmolin RJ , Yan M , Tian F , Chen X , Chen G and Cao W (2015) Prediction of long noncoding RNA functions with co‐expression network in esophageal squamous cell carcinoma. BMC Cancer 15, 168. 25885227 10.1186/s12885-015-1179-z PMC4377028

[mol212683-bib-0014] Hu X , Hong Y and Shang C (2019) Knockdown of long non‐coding RNA SNHG5 inhibits malignant cellular phenotypes of glioma via Wnt/CTNNB1 signaling pathway. J Cancer 10, 1333–1340. 30854143 10.7150/jca.29517 PMC6400671

[mol212683-bib-0015] Huarte M and Rinn JL (2010) Large non‐coding RNAs: missing links in cancer? Hum Mol Genet 19, R152–161. 20729297 10.1093/hmg/ddq353 PMC2953740

[mol212683-bib-0016] Jemal A , Bray F , Center MM , Ferlay J , Ward E and Forman D (2011) Global cancer statistics. CA Cancer J Clin 61, 69–90. 21296855 10.3322/caac.20107

[mol212683-bib-0017] Jin XJ , Chen XJ , Zhang ZF , Hu WS , Ou RY , Li S , Xue JS , Chen LL , Hu Y and Zhu H (2019) Long noncoding RNA SNHG12 promotes the progression of cervical cancer via modulating miR‐125b/STAT3 axis. J Cell Physiol 234, 6624–6632. 30246459 10.1002/jcp.27403

[mol212683-bib-0018] Krishnamurthy N and Kurzrock R (2018) Targeting the Wnt/beta‐catenin pathway in cancer: Update on effectors and inhibitors. Cancer Treat Rev 62, 50–60. 29169144 10.1016/j.ctrv.2017.11.002 PMC5745276

[mol212683-bib-0019] Le PN , Keysar SB , Miller B , Eagles JR , Chimed TS , Reisinger J , Gomez KE , Nieto C , Jackson BC , Somerset HL *et al* (2019) Wnt signaling dynamics in head and neck squamous cell cancer tumor‐stroma interactions. Mol Carcinog 58, 398–410. 30378175 10.1002/mc.22937 PMC6460915

[mol212683-bib-0020] Li DJ , Shi M and Wang Z (2016) RUNX3 reverses cisplatin resistance in esophageal squamous cell carcinoma via suppression of the protein kinase B pathway. Thoracic Cancer 7, 570–580. 27766776 10.1111/1759-7714.12370 PMC5129150

[mol212683-bib-0021] Li XG , Wang Z , Chen RQ , Fu HL , Gao CQ , Yan HC , Xing GX and Wang XQ . (2018a). LGR5 and BMI1 Increase Pig Intestinal Epithelial Cell Proliferation by Stimulating WNT/beta‐Catenin Signaling. Int J Mol Sci 19, E1036 10.3390/ijms19041036. 29601474 PMC5979389

[mol212683-bib-0022] Li X , Wu Z , Mei Q , Li X , Guo M , Fu X and Han W (2013) Long non‐coding RNA HOTAIR, a driver of malignancy, predicts negative prognosis and exhibits oncogenic activity in oesophageal squamous cell carcinoma. Br J Cancer 109, 2266–2278. 24022190 10.1038/bjc.2013.548 PMC3798955

[mol212683-bib-0023] Li Y , Zeng C , Hu J , Pan Y , Shan Y , Liu B and Jia L (2018b) Long non‐coding RNA‐SNHG7 acts as a target of miR‐34a to increase GALNT7 level and regulate PI3K/Akt/mTOR pathway in colorectal cancer progression. J Hematol Oncol 11, 89. 29970122 10.1186/s13045-018-0632-2 PMC6029165

[mol212683-bib-0024] Liang H , Yu T , Han Y , Jiang H , Wang C , You T , Zhao X , Shan H , Yang R , Yang L *et al* (2018a) LncRNA PTAR promotes EMT and invasion‐metastasis in serous ovarian cancer by competitively binding miR‐101‐3p to regulate ZEB1 expression. Mol Cancer 17, 119. 30098599 10.1186/s12943-018-0870-5 PMC6087007

[mol212683-bib-0025] Liang Y , Chen X , Wu Y , Li J , Zhang S , Wang K , Guan X , Yang K and Bai Y (2018b) LncRNA CASC9 promotes esophageal squamous cell carcinoma metastasis through upregulating LAMC2 expression by interacting with the CREB‐binding protein. Cell Death Differ 25, 1980–1995. 29511340 10.1038/s41418-018-0084-9 PMC6219493

[mol212683-bib-0026] Lin C , Wang Y , Wang Y , Zhang S , Yu L , Guo C and Xu H (2017) Transcriptional and posttranscriptional regulation of HOXA13 by lncRNA HOTTIP facilitates tumorigenesis and metastasis in esophageal squamous carcinoma cells. Oncogene 36, 5392–5406. 28534516 10.1038/onc.2017.133

[mol212683-bib-0027] Liu Y , Zhou J , Wang S , Song Y , Zhou J and Ren F (2019) Long non‐coding RNA SNHG12 promotes proliferation and invasion of colorectal cancer cells by acting as a molecular sponge of microRNA‐16. Exp Therap Med 18, 1212–1220. 31316616 10.3892/etm.2019.7650 PMC6601377

[mol212683-bib-0028] Lv XB , Lian GY , Wang HR , Song E , Yao H and Wang MH (2013) Long noncoding RNA HOTAIR is a prognostic marker for esophageal squamous cell carcinoma progression and survival. PLoS ONE 8, e63516. 23717443 10.1371/journal.pone.0063516 PMC3662674

[mol212683-bib-0029] Ma L , Bajic VB and Zhang Z (2013) On the classification of long non‐coding RNAs. RNA Biol 10, 925–933. 23696037 10.4161/rna.24604 PMC4111732

[mol212683-bib-0030] Miyazaki T , Kato H , Fukuchi M , Nakajima M and Kuwano H (2003) EphA2 overexpression correlates with poor prognosis in esophageal squamous cell carcinoma. Int J Cancer 103, 657–663. 12494475 10.1002/ijc.10860

[mol212683-bib-0031] Reya T and Clevers H (2005) Wnt signalling in stem cells and cancer. Nature 434, 843–850. 15829953 10.1038/nature03319

[mol212683-bib-0032] Rustgi AK and El‐Serag HB (2014) Esophageal carcinoma. New Engl J Med 371, 2499–2509. 25539106 10.1056/NEJMra1314530

[mol212683-bib-0033] Shao Q , Xu J , Deng R , Wei W , Zhou B , Yue C , Zhu M and Zhu H (2019) SNHG 6 promotes the progression of Colon and Rectal adenocarcinoma via miR‐101‐3p and Wnt/beta‐catenin signaling pathway. BMC Gastroenterol 19, 163. 31533634 10.1186/s12876-019-1080-3 PMC6749705

[mol212683-bib-0034] Song J , Wu X , Ma R , Miao L , Xiong L and Zhao W (2019) Long noncoding RNA SNHG12 promotes cell proliferation and activates Wnt/beta‐catenin signaling in prostate cancer through sponging microRNA‐195. J Cell Biochem 120, 13066–13075. 30945357 10.1002/jcb.28578

[mol212683-bib-0035] Tang Y , Yang P , Zhu Y and Su Y (2020) LncRNA TUG1 contributes to ESCC progression via regulating miR‐148a‐3p/MCL‐1/Wnt/β‐catenin axis in vitro. Thorac Cancer 11, 82–94. 31742924 10.1111/1759-7714.13236 PMC6938768

[mol212683-bib-0036] Wang F , Zhu W , Yang R , Xie W and Wang D (2019a) LncRNA ZEB2‐AS1 contributes to the tumorigenesis of gastric cancer via activating the Wnt/beta‐catenin pathway. Mol Cell Biochem 456, 73–83. 30635820 10.1007/s11010-018-03491-7

[mol212683-bib-0037] Wang H , Wang G , Gao Y , Zhao C , Li X , Zhang F , Jiang C and Wu B (2018) Lnc‐SNHG1 Activates the TGFBR2/SMAD3 and RAB11A/Wnt/beta‐Catenin Pathway by Sponging MiR‐302/372/373/520 in Invasive Pituitary Tumors. Cell Physiol Biochem 48, 1291–1303. 30048990 10.1159/000492089

[mol212683-bib-0038] Wang L , Yu X , Zhang Z , Pang L , Xu J , Jiang J , Liang W , Chai Y , Hou J and Li F (2017a) Linc‐ROR promotes esophageal squamous cell carcinoma progression through the derepression of SOX9. J Exp Clin Cancer Res 36, 182. 29237490 10.1186/s13046-017-0658-2 PMC5727696

[mol212683-bib-0039] Wang MW , Liu J , Liu Q , Xu QH , Li TF , Jin S and Xia TS (2017b) LncRNA SNHG7 promotes the proliferation and inhibits apoptosis of gastric cancer cells by repressing the P15 and P16 expression. Eur Rev Med Pharmacol Sci 21, 4613–4622. 29131253

[mol212683-bib-0040] Wang Y , Liang S , Yu Y , Shi Y and Zheng H (2019b) Knockdown of SNHG12 suppresses tumor metastasis and epithelial‐mesenchymal transition via the Slug/ZEB2 signaling pathway by targeting miR‐218 in NSCLC. Oncol Lett 17, 2356–2364. 30719111 10.3892/ol.2018.9880 PMC6351734

[mol212683-bib-0041] Wei GH and Wang X (2017) lncRNA MEG3 inhibit proliferation and metastasis of gastric cancer via p53 signaling pathway. Eur Rev Med Pharmacol Sci 21, 3850–3856. 28975980

[mol212683-bib-0042] Wu Y , Hu L , Liang Y , Li J , Wang K , Chen X , Meng H , Guan X , Yang K and Bai Y (2017) Up‐regulation of lncRNA CASC9 promotes esophageal squamous cell carcinoma growth by negatively regulating PDCD4 expression through EZH2. Mol Cancer 16, 150. 28854977 10.1186/s12943-017-0715-7 PMC5577767

[mol212683-bib-0043] Xiao Y , Pan J , Geng Q and Wang G (2019) LncRNA MALAT1 increases the stemness of gastric cancer cells via enhancing SOX2 mRNA stability. FEBS Open Bio 9, 1212–1222. 10.1002/2211-5463.12649 PMC6609564 31037832

[mol212683-bib-0044] Xu LJ , Yu XJ , Wei B , Hui HX , Sun Y , Dai J and Chen XF (2018a) LncRNA SNHG7 promotes the proliferation of esophageal cancer cells and inhibits its apoptosis. Eur Rev Med Pharmacol Sci 22, 2653–2661. 29771415 10.26355/eurrev_201805_14961

[mol212683-bib-0045] Xu M , Chen X , Lin K , Zeng K , Liu X , Pan B , Xu X , Xu T , Hu X , Sun L *et al* (2018b) The long noncoding RNA SNHG1 regulates colorectal cancer cell growth through interactions with EZH2 and miR‐154‐5p. Mol Cancer 17, 141. 30266084 10.1186/s12943-018-0894-x PMC6162892

[mol212683-bib-0046] Yan Y , Fan Q , Wang L , Zhou Y , Li J and Zhou K (2017) LncRNA Snhg1, a non‐degradable sponge for miR‐338, promotes expression of proto‐oncogene CST3 in primary esophageal cancer cells. Oncotarget 8, 35750–35760. 28423738 10.18632/oncotarget.16189 PMC5482614

[mol212683-bib-0047] Yang S , Liu Y , Li M‐Y , Ng CSH , Yang S‐L , Wang S , Zou C , Dong Y , Du J , Long X *et al* (2017) FOXP3 promotes tumor growth and metastasis by activating Wnt/β‐catenin signaling pathway and EMT in non‐small cell lung cancer. Mol Cancer 16, 124–124. 28716029 10.1186/s12943-017-0700-1 PMC5514503

[mol212683-bib-0048] Yu F , Zhou C , Zeng H , Liu Y and Li S (2018) BMI1 activates WNT signaling in colon cancer by negatively regulating the WNT antagonist IDAX. Biochem Biophys Res Comm 496, 468–474. 29337063 10.1016/j.bbrc.2018.01.063

[mol212683-bib-0049] Yuan S‐X , Wang J , Yang F , Tao Q‐F , Zhang J , Wang L‐L , Yang Y , Liu H , Wang Z‐G , Xu Q‐G *et al* (2016) Long noncoding RNA DANCR increases stemness features of hepatocellular carcinoma by derepression of CTNNB1. Hepatology 63, 499–511. 25964079 10.1002/hep.27893

[mol212683-bib-0050] Zhang M , Zhao Y , Zhang Y , Wang D , Gu S , Feng W , Peng W , Gong A and Xu M (2018) LncRNA UCA1 promotes migration and invasion in pancreatic cancer cells via the Hippo pathway. Biochimica et biophysica acta. Mol Basis Dis 1864, 1770–1782. 10.1016/j.bbadis.2018.03.005 29510195

[mol212683-bib-0051] Zhang Y , Jin X , Wang Z , Zhang X , Liu S and Liu G (2017) Downregulation of SNHG1 suppresses cell proliferation and invasion by regulating Notch signaling pathway in esophageal squamous cell cancer. Cancer Biomark 21, 89–96. 29081407 10.3233/CBM-170286 PMC13075759

[mol212683-bib-0052] Zhao L , Sun H , Kong H , Chen Z , Chen B and Zhou M (2017) The Lncrna‐TUG1/EZH2 Axis Promotes Pancreatic Cancer Cell Proliferation, Migration and EMT Phenotype Formation Through Sponging Mir‐382. Cell Physiol Biochem 42, 2145–2158. 28813705 10.1159/000479990

