# Supplementary figures and images for: Long noncoding RNA SNHG12 induces proliferation, migration, epithelial–mesenchymal transition, and stemness of esophageal squamous cell carcinoma cells via post‐transcriptional regulation of BMI1 and CTNNB1
Source: Mol Oncol. 2020 Jun 18;14(9):2332–51. doi: 10.1002/1878-0261.12683 (PMC7463312; doi:10.1002/1878-0261.12683)

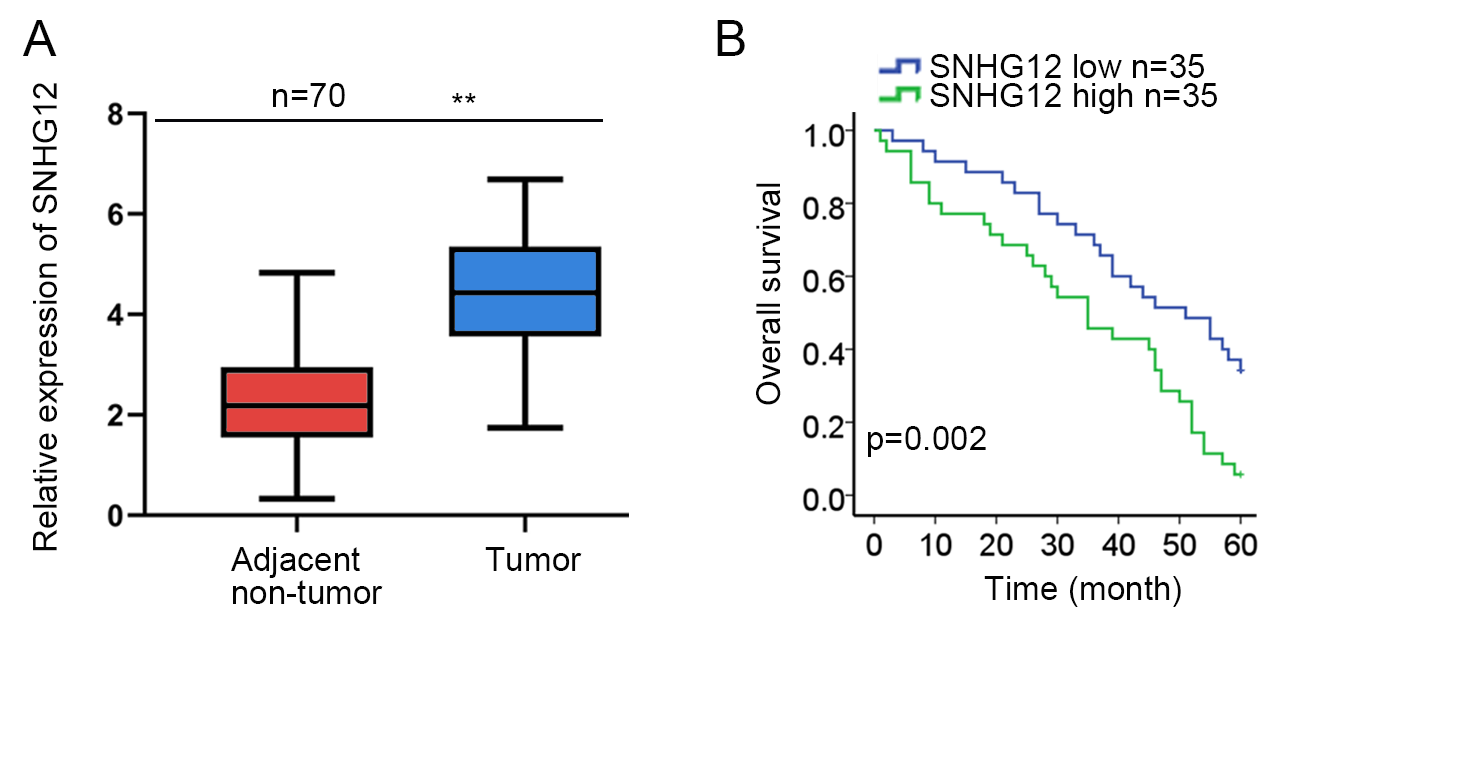

Supplement: Supplementary file 1 — Fig. S1. SNHG12 level in ESCC specimens and its prognostic value. (A) qRT‐PCR of SNHG12 level in ESCC specimens versus paired para‐tumor tissues (n = 5; Paired student’s t‐test). (B) Kaplan–Meier analysis of correlation between SNHG12 level and overall survival in ESCC patients (log‐rank test). Results were all exhibited as the mean ± Standard Deviation (SD) and taken from more than three independent experiments. **P < 0.01. [file MOL2-14-2332-s001.tif]

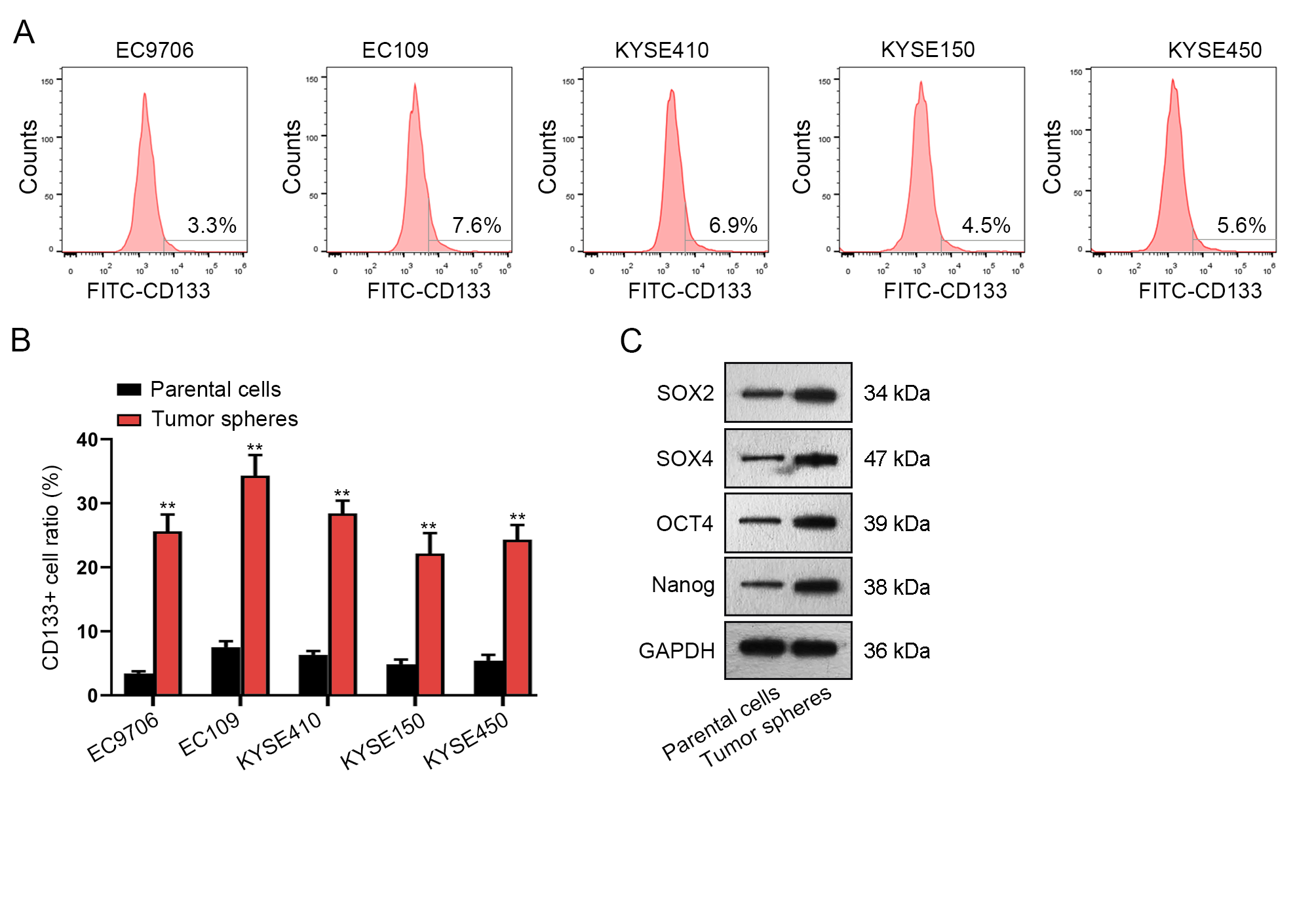

Supplement: Supplementary file 2 — Fig. S2. Sorting and enriching of CD133+ ESCC cells. (A) CD133+ ESCC cells were sorted by flow cytometry analysis. (B) Sphere formation was used to enrich CD133+ ESCC cells, and CD133+ ratio in tumorspheres derived by ESCC cells versus the parental cells was analyzed by flow cytometry (n = 5; Student’s t‐test). (C) Western blot of stemness specific genes in tumorspheres derived by ESCC cells versus the parental cells (n = 5). Results were all exhibited as the mean ± Standard Deviation (SD) and taken from more than three independent experiments. **P < 0.01. [file MOL2-14-2332-s002.tif]

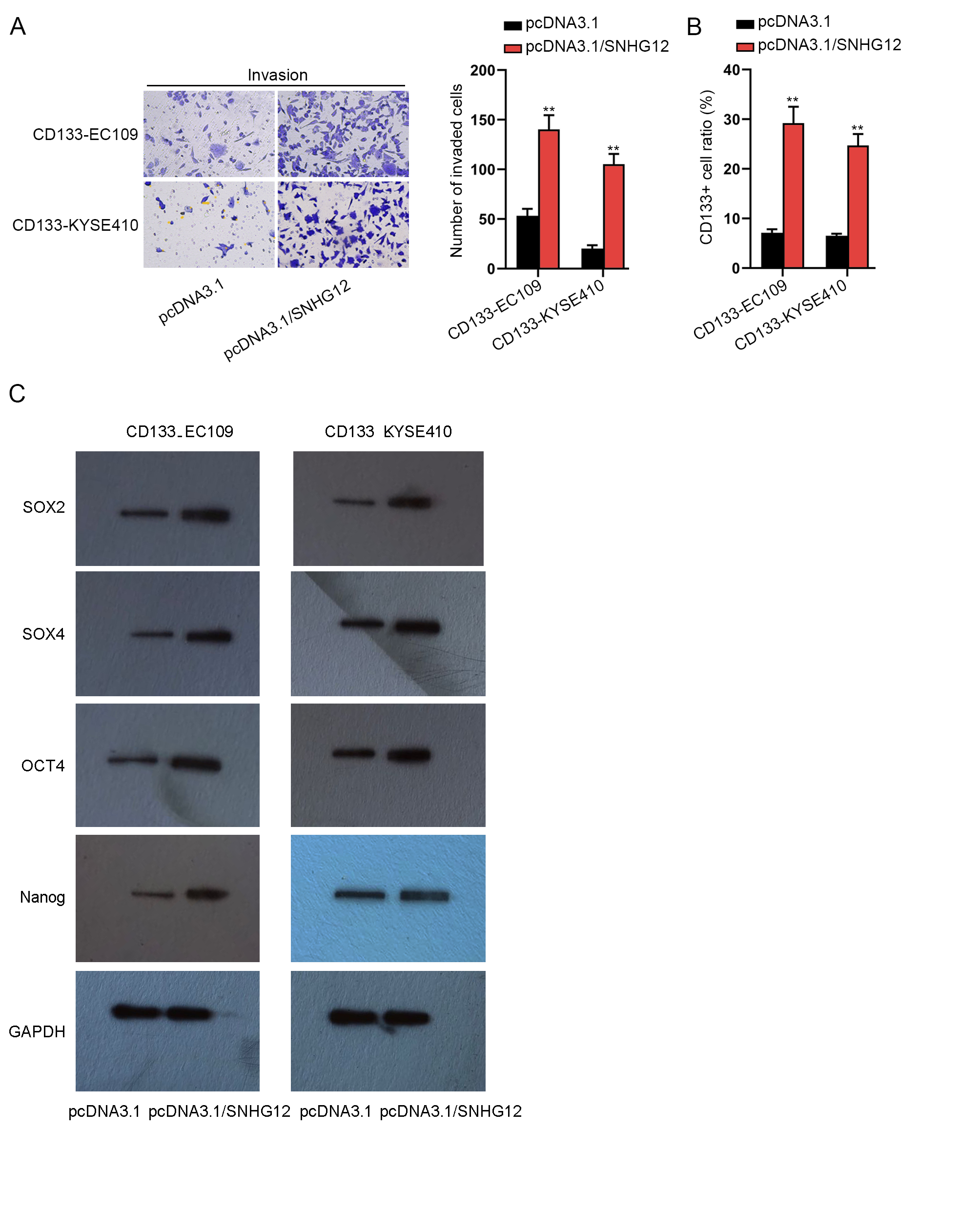

Supplement: Supplementary file 3 — Fig. S3. Effect of SNHG12 overexpression on cell invasion and CD133+ ratio. (A) Pictures (bar value = 100 μm) of invasive ESCC cells in transwell system under SNHG12 overexpression and the number of cells per field was quantified (n = 5; Student’s t‐test). (B) CD133+ ratio in ESCC cells with SNHG12 overexpression was quantified by flow cytometry analysis (n = 5; Student’s t‐test). (C) Original data of western blot in Figure 2G (n = 5). Results were all exhibited as the mean ± Standard Deviation (SD) and taken from more than three independent experiments. **P < 0.01. [file MOL2-14-2332-s003.tif]

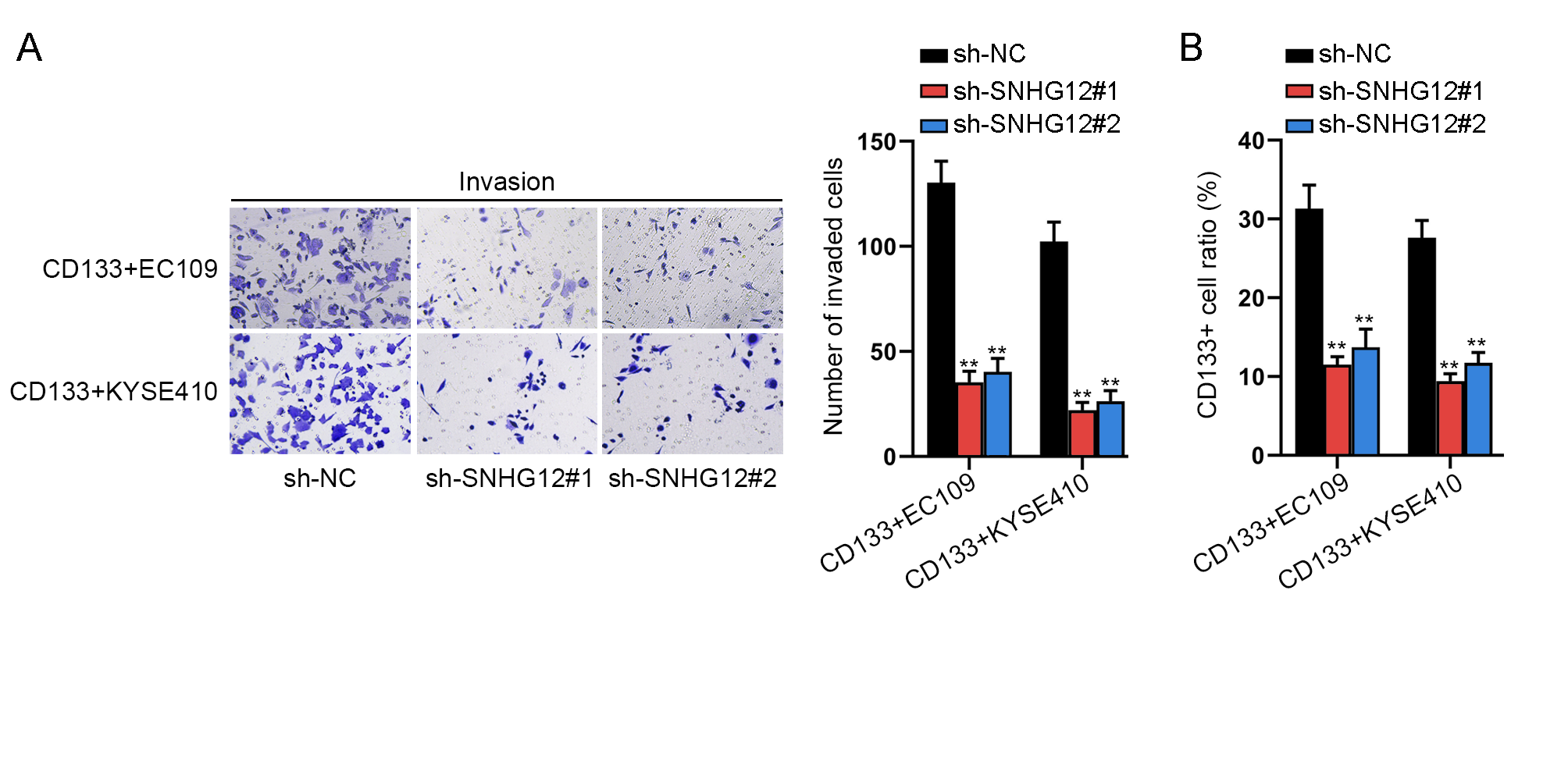

Supplement: Supplementary file 4 — Fig. S4. Effects of SNHG12 knockdown on cell invasion and CD133+ ratio. (A) Pictures (bar value = 100 μm) of invasive ESCC cells in transwell system under SNHG12 knockdown and the number of cells per field was quantified (n = 5; one‐way ANOVA). (B) CD133+ ratio in ESCC cells with SNHG12 knockdown was quantified by flow cytometry analysis (n = 5; one‐way ANOVA). Results were all exhibited as the mean ± Standard Deviation (SD) and taken from more than three independent experiments. **P < 0.01. [file MOL2-14-2332-s004.tif]

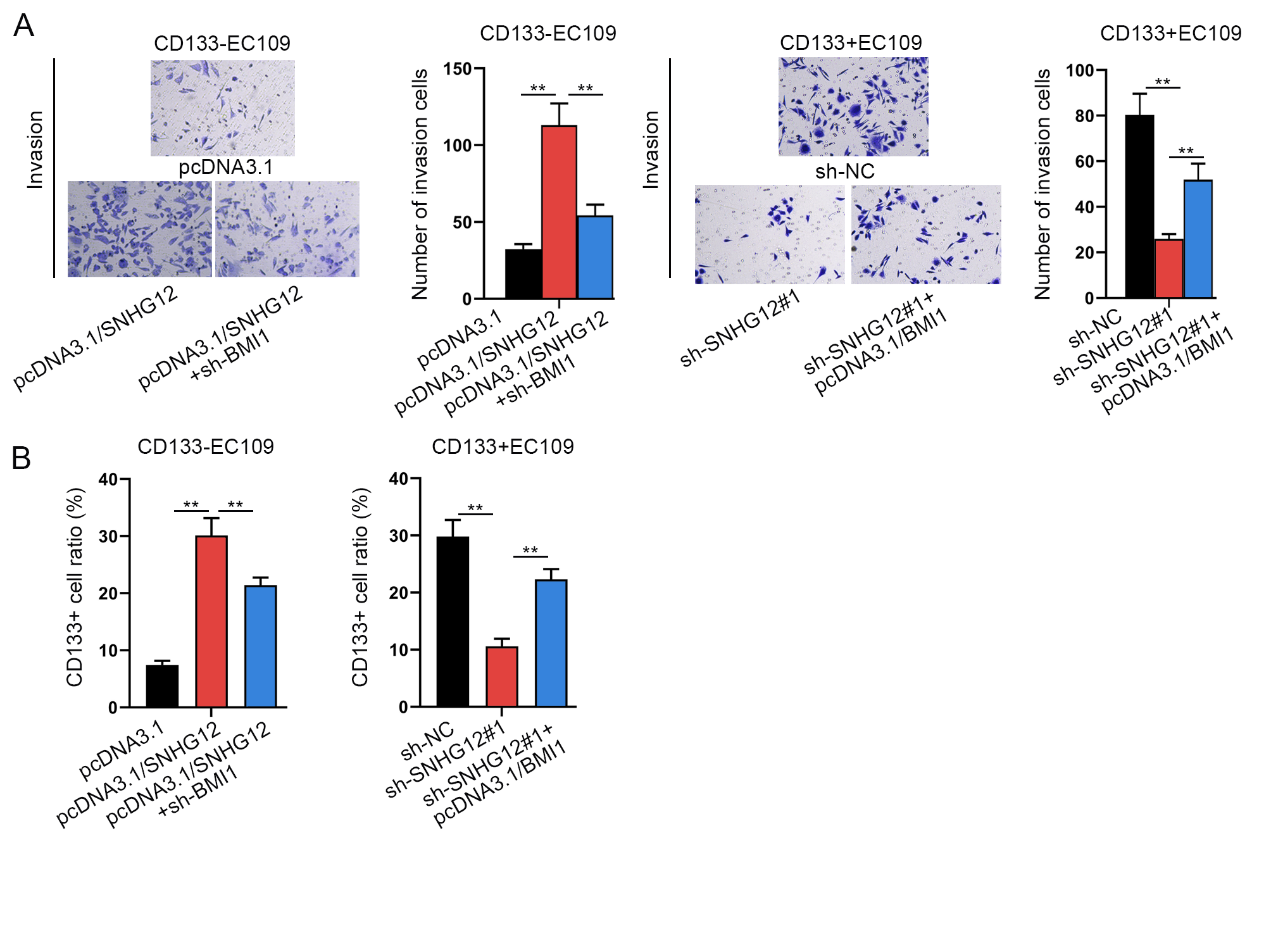

Supplement: Supplementary file 5 — Fig. S5. BMI1 rescued SNHG12 function in cell invasion and CD133+ ratio in ESCC. (A) Pictures (bar value = 100 μm) of invasive ESCC cells in transwell system with indicated transfections and the number of cells per field was quantified (n = 5; one‐way ANOVA). (B) CD133+ ratio in ESCC cells with indicated transfections was quantified by flow cytometry analysis (n = 5; one‐way ANOVA). Results were all exhibited as the mean ± Standard Deviation (SD) and taken from more than three independent experiments. **P < 0.01. [file MOL2-14-2332-s005.tif]

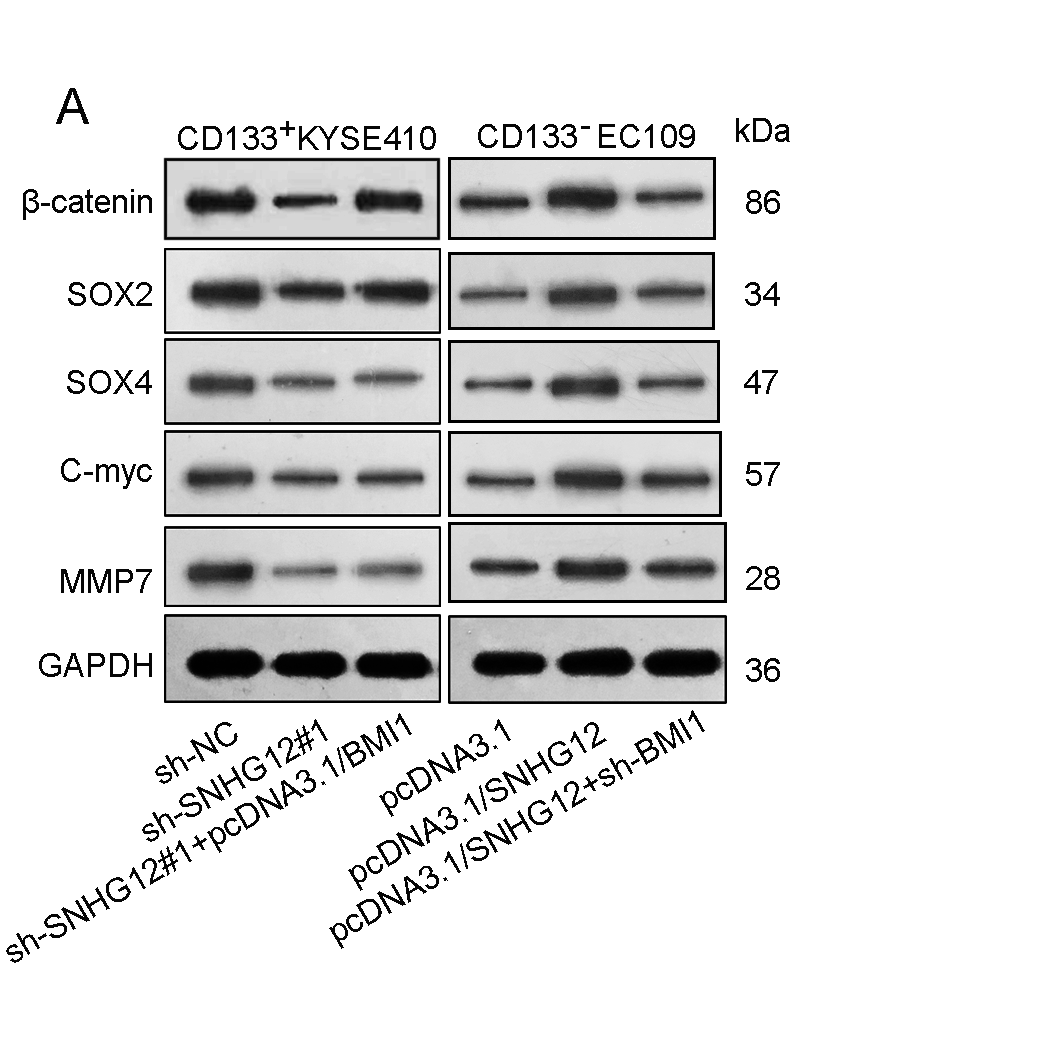

Supplement: Supplementary file 6 — Fig. S6. Western blot of key factors in Wnt pathway. (A) Western blot assay measured the Wnt pathway‐related protein level in different transfected groups (n = 5). Results were all exhibited as the mean ± Standard Deviation (SD) and taken from more than three independent experiments. [file MOL2-14-2332-s006.tif]

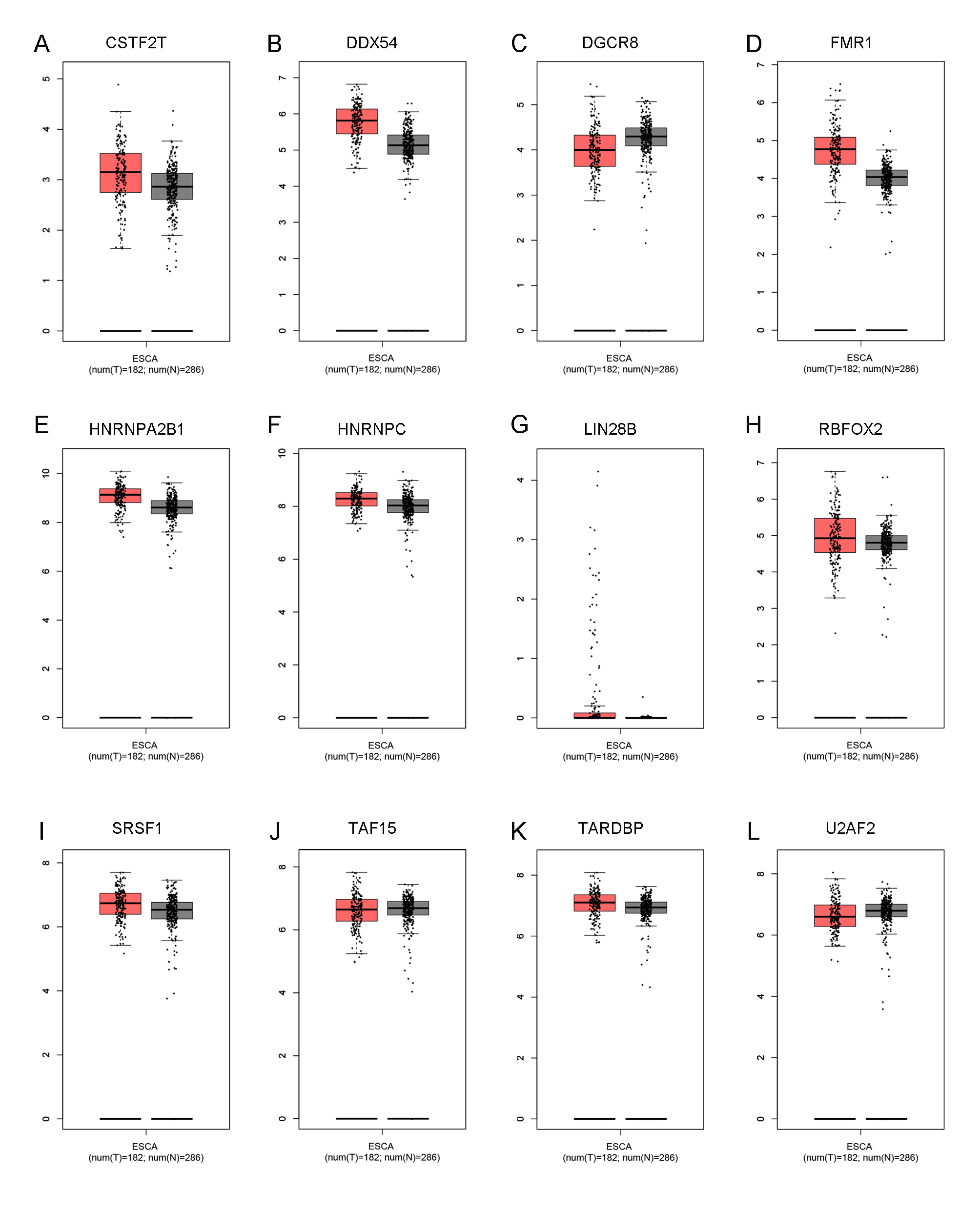

Supplement: Supplementary file 7 — Fig. S7. Analysis of RBP expressions in ESCA samples from GEPIA. (A‐L) The expression of the expression of other twelve RBPs in ESCA (esophageal carcinoma) tissues and corresponding normal tissues was obtained from TCGA database. Results were all exhibited as the mean ± Standard Deviation (SD) and taken from more than three independent experiments. [file MOL2-14-2332-s007.tif]

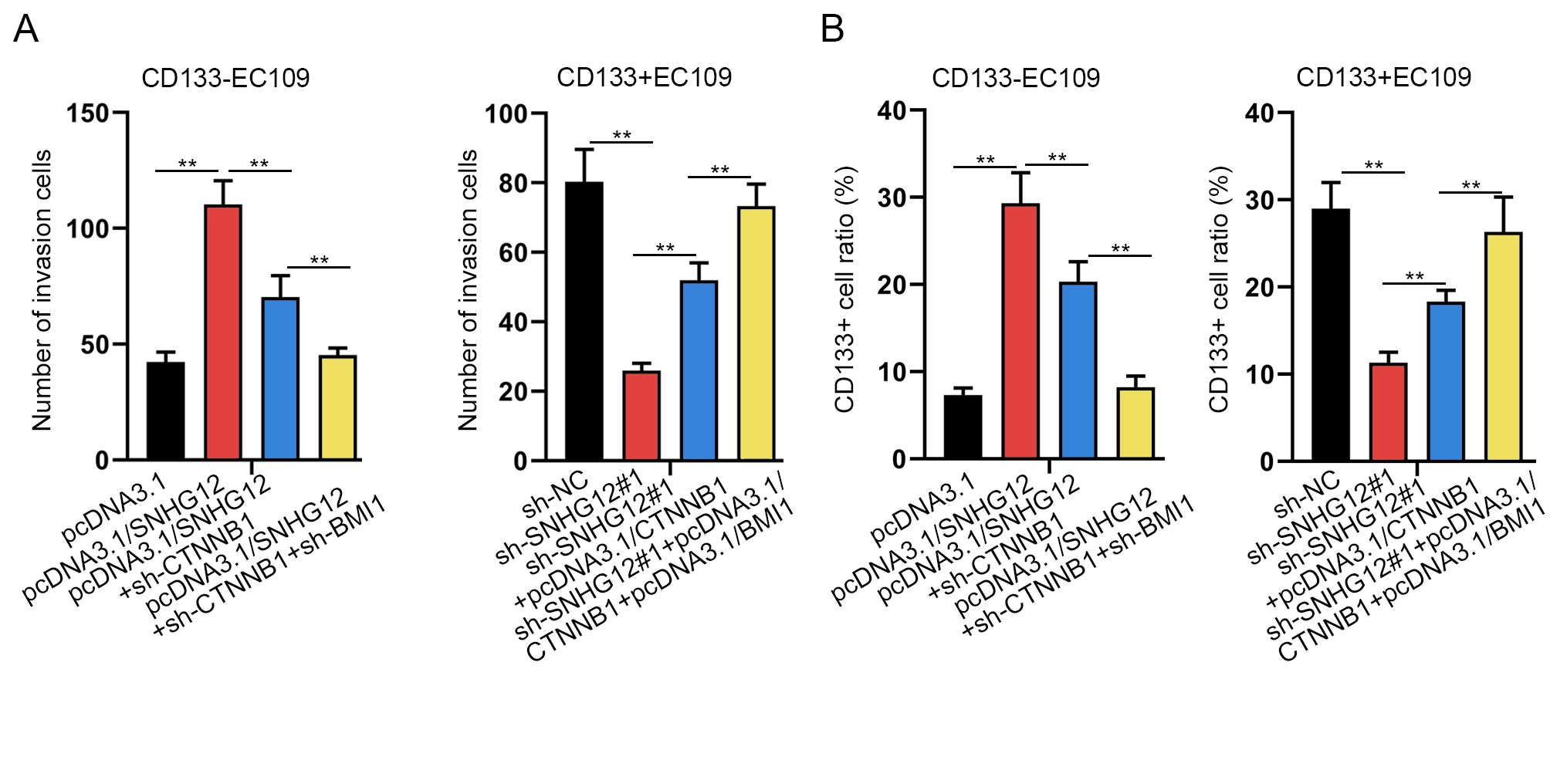

Supplement: Supplementary file 8 — Fig. S8. BMI1 and CTNNB1 rescue SNHG12 function in cell invasion and CD133+ ratio in ESCC. (A) Number of invasive ESCC cells in transwell system with indicated transfections per field was quantified (n = 5; one‐way ANOVA). (B) CD133+ ratio in ESCC cells with indicated transfections was quantified by flow cytometry analysis (n = 5; one‐way ANOVA). Results were all exhibited as the mean ± Standard Deviation (SD) and taken from more than three independent experiments. **P < 0.01. [file MOL2-14-2332-s008.tif]

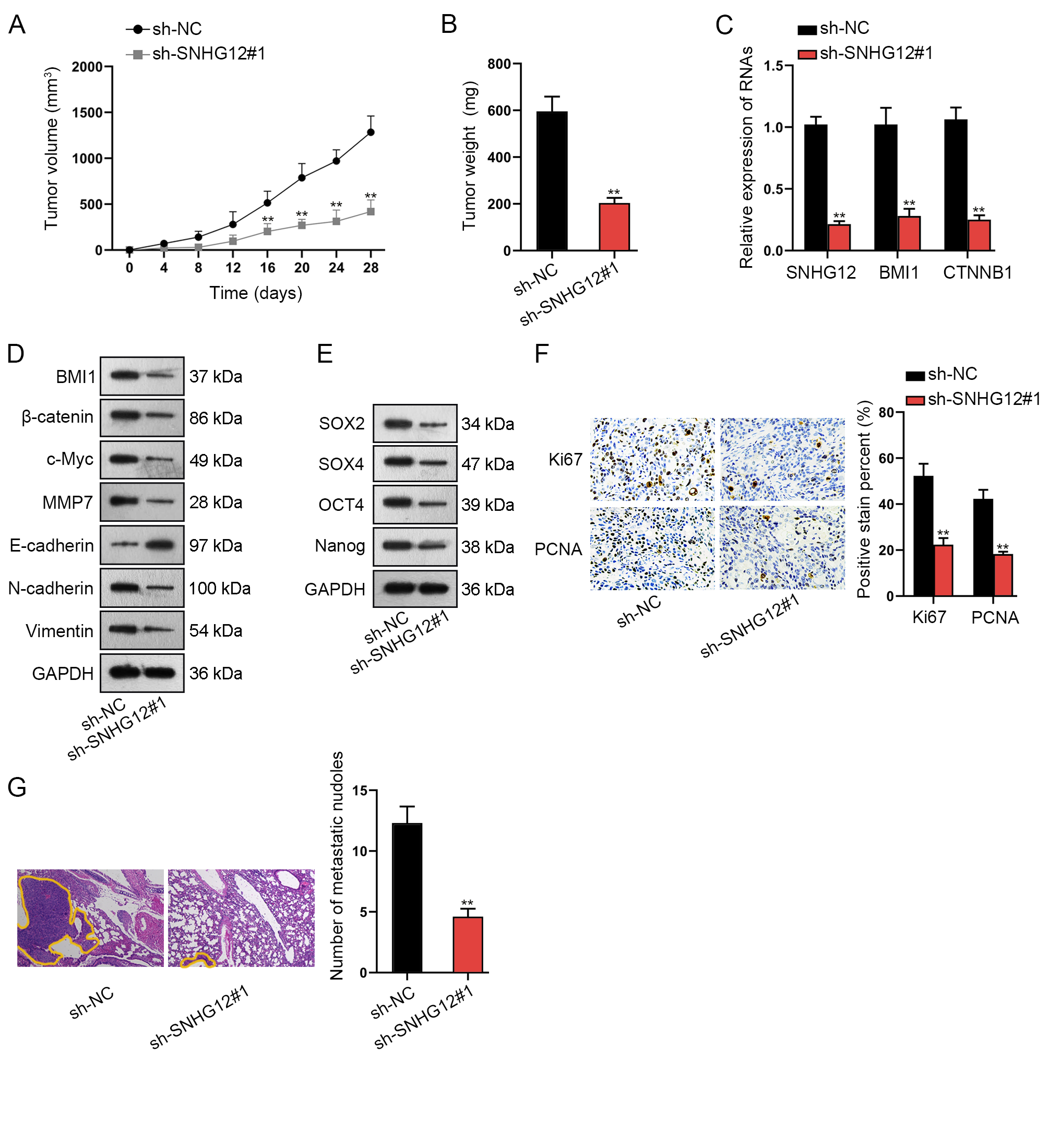

Supplement: Supplementary file 9 — Fig. S9. Function of SNHG12 in vivo. (A) Growth curve of xenografts in mice subcutaneously injected with CD133+ EC109 cells with sh‐NC or sh‐SNHG12#1 (n = 5; Student’s t‐test). (B) Tumor weight at day 28 after injection was detected (n = 5; Student’s t‐test). (C) qRT‐PCR of SNHG12, BMI1, and CTNNB1 levels in xenografts of each group (n = 5; Student’s t‐test). (D‐E) Western blots of BMI1, β‐catenin, c‐Myc, MMP7, E‐cadherin, N‐cadherin, Vimentin, SOX2, SOX4, OCT4, and Nanog in xenografts of each group (n = 5). (F) IHC picture of Ki‐67 and PCNA in xenografts of each group. Scale bar: 100 μm (n = 5; Student’s t‐test). (G) Pictures and quantification of HE staining of metastatic nodules in mice intravenously injected with CD133+ EC109 cells with sh‐NC or sh‐SNHG12#1. (Scale bar: 100 μm; n = 5; Student’s t‐test). Results were all exhibited as the mean ± Standard Deviation (SD) and taken from more than three independent experiments. **P < 0.01. [file MOL2-14-2332-s009.tif]

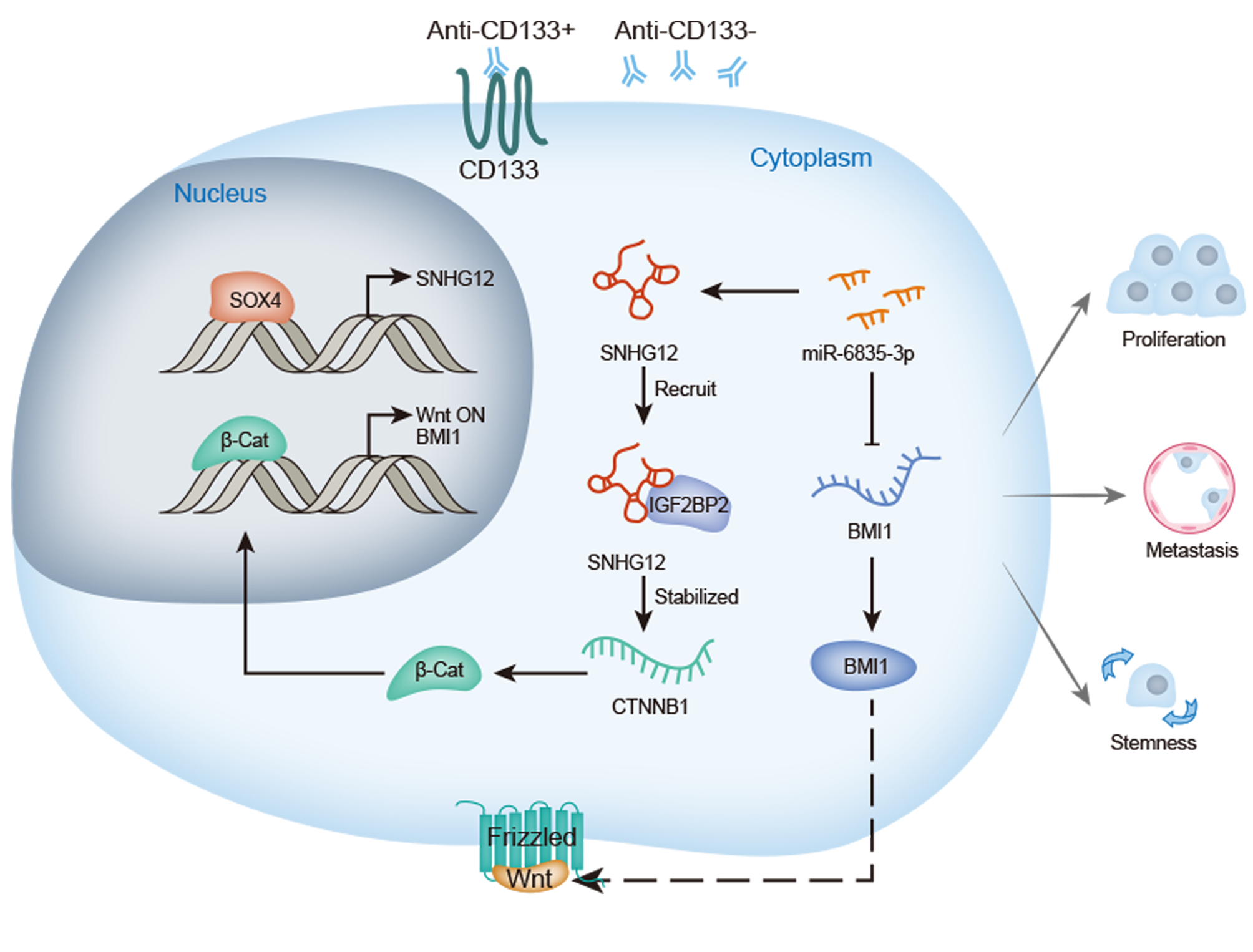

Supplement: Supplementary file 10 — Fig. S10. In esophageal squamous cell carcinoma, SNHG12 regulates BMI1 expression via sponging miR‐6835‐3p, and enhances CTNNB1 stability via recruiting IGF2BP2. Thus, SNHG12 activates Wnt/β‐catenin signaling and facilitates proliferation, metastasis and stemness. Downstream of the Wnt pathway, SOX4 binds with SNHG12 promoter to transcriptionally activate SNHG12. [file MOL2-14-2332-s010.tif]
